# Molecular mechanisms of inorganic-phosphate release from the core and barbed end of actin filaments

**DOI:** 10.1038/s41594-023-01101-9

**Published:** 2023-09-25

**Authors:** Wout Oosterheert, Florian E. C. Blanc, Ankit Roy, Alexander Belyy, Micaela Boiero Sanders, Oliver Hofnagel, Gerhard Hummer, Peter Bieling, Stefan Raunser

**Affiliations:** 1 Department of Structural Biochemistry, Max Planck Institute of Molecular Physiology, Dortmund, Germany; 2 Department of Theoretical Biophysics, Max Planck Institute of Biophysics, Frankfurt am Main, Germany; 3 Department of Systemic Cell Biology, Max Planck Institute of Molecular Physiology, Dortmund, Germany; 4 Institute for Biophysics, Goethe University, Frankfurt am Main, Germany

**Keywords:** Cryoelectron microscopy, Molecular biophysics, Cytoskeletal proteins

## Abstract

The release of inorganic phosphate (P_i_) from actin filaments constitutes a key step in their regulated turnover, which is fundamental to many cellular functions. The mechanisms underlying P_i_ release from the core and barbed end of actin filaments remain unclear. Here, using human and bovine actin isoforms, we combine cryo-EM with molecular-dynamics simulations and in vitro reconstitution to demonstrate how actin releases P_i_ through a ‘molecular backdoor’. While constantly open at the barbed end, the backdoor is predominantly closed in filament-core subunits and opens only transiently through concerted amino acid rearrangements. This explains why P_i_ escapes rapidly from the filament end but slowly from internal subunits. In a nemaline-myopathy-associated actin variant, the backdoor is predominantly open in filament-core subunits, resulting in accelerated P_i_ release and filaments with drastically shortened ADP-P_i_ caps. Our results provide the molecular basis for P_i_ release from actin and exemplify how a disease-linked mutation distorts the nucleotide-state distribution and atomic structure of the filament.

## Abstract (web summary)

Release of inorganic phosphate (P_i_) from actin marks older actin filaments for disassembly. Here, the authors show how P_i_ exits the F-actin interior through a ‘molecular backdoor’. The backdoor arrangement is distorted in a disease-linked actin variant.

## Main

The dynamic turnover of actin filaments (F-actin) controls the shape and movement of eukaryotic cells and is driven by changes in the molecular identity of the adenine nucleotide that is bound to actin^1–3^. Monomeric actin (G-actin) displays only weak ATPase activity^4^, but actin polymerization results in the flattening of the protein and a rearrangement of amino acids and water molecules near the nucleotide-binding site^5–8^. Accordingly, the ATPase activity of actin increases by about 42,000-fold, and hydrolysis takes place within seconds of filament formation (at a rate of 0.3 s^−1^) (ref. ^9^). Because the release of cleaved P_i_ occurs at much slower rates than does ATP hydrolysis^10^, the cap of a growing filament is generally rich in ADP-P_i_-bound subunits^11^, whereas ‘aged’ F-actin primarily adopts the ADP-bound state. In vivo, these changes in the nucleotide state are sensed by several actin-binding proteins (ABPs)^12–14^. For instance, ADF/cofilin family proteins efficiently bind and sever the ADP-bound state of the filament, but bind only weakly to ADP-P_i_–F-actin^15–17^. Severing by ADF/cofilin promotes actin turnover following network assembly^18^, making P_i_ release from the F-actin interior an essential mechanism for polarized, directed cell migration.

It was long debated whether the P_i_ release rate of a given F-actin subunit depends on the nucleotide state of its neighboring subunits^10,19,20^. Finally, single-filament experiments showed that P_i_ release from the F-actin core is stochastic^21,22^; each ADP-P_i_-bound subunit in the filament releases its bound phosphate with equal probability at a rate of 0.002 to 0.007 s^−1^, which corresponds to a half-time (*τ*) of several minutes^10,21–25^. During filament growth, P_i_ release occurs solely from filament core subunits because the barbed-end growth velocity (~10–500 monomers s^−1^) (ref. ^26^) is much faster than the ATP hydrolysis rate of actin (0.3 s^−1^), indicating that barbed-end subunits effectively adopt the ATP state only before becoming internal subunits. However, after the transition from filament growth to depolymerization, actin subunits that have hydrolyzed ATP and adopt the ADP-P_i_ state can be exposed at the shortening barbed end. Interestingly, these barbed-end subunits release P_i_ more than 300-fold faster (~2 s^−1^) than do those within the filament core, even though the affinities for P_i_ binding to the barbed end and the filament core are essentially the same^21,27^.

At the atomic level, P_i_ release from actin was investigated in pioneering molecular dynamics (MD) simulation studies in the late 1990s (refs. ^28,29^), which suggested that the disruption of the ionic bond between P_i_ and the nucleotide-associated divalent cation (Mg^2+^ or Ca^2+^) could represent the rate-limiting step for P_i_ release, because actin rearrangements were not required for P_i_ to exit. These studies furthermore predicted that P_i_ escapes through an open ‘backdoor’ in the actin molecule, with residues R177 and methylated H73 potentially being mediators of P_i_ release. However, a central limitation of the simulations was that they were performed on a G-actin structure^30^, because high-resolution F-actin structures were unavailable at the time. The first sub-4-Å cryogenic electron microscopy (cryo-EM) structures of F-actin revealed that, within the flattened conformation of the filament, P_i_ cannot freely diffuse from the F-actin interior^31^; R177 participates in a hydrogen bonding network with the side chain of N111 and the backbones of H73 and G74, forming a closed backdoor conformation in F-actin. Accordingly, recently published cryo-EM structures of F-actin bound to ADP-P_i_ and ADP at resolutions beyond 2.5 Å showed a closed backdoor in both the pre- and post-release states^8,32^. This suggests that P_i_ escapes the filament interior when F-actin is in a transient, high-energy state that requires substantial rearrangements and that is difficult to capture using static imaging techniques such as cryo-EM. In a recent model of P_i_ release, a rotameric switch of residue S14 from a hydrogen bond interaction with the backbone amide of G74 to that of G158 would enable P_i_ to approach the R177-N111 backdoor and egress^33^. However, in the absence of experimental validation, the molecular principles of P_i_ release from F-actin remain elusive, and further evidence is required to determine whether P_i_ exits the filament interior through the postulated backdoor or another route. Additionally, the structural basis for why P_i_ release from actin subunits at the barbed end is orders of magnitude faster than from core subunits is still unknown.

Here, we uncover that P_i_ release from filament core and barbed-end subunits occurs through a common backdoor. For internal subunits, the backdoor is predominantly closed and its transient opening is kinetically limiting, whereas at the barbed end, the backdoor is open and P_i_ can escape from the ultimate actin subunit without large rearrangements in the protein. Strikingly, we also characterize an actin disease variant (p.N111S) that adopts an open backdoor arrangement in internal subunits and hence releases P_i_ without considerable delay. Our results provide a detailed molecular description of P_i_ release from F-actin and outline a general approach of studying disease-linked actin variants.

## Results

### An open backdoor in the ultimate barbed-end subunit

**Fig. 1:**
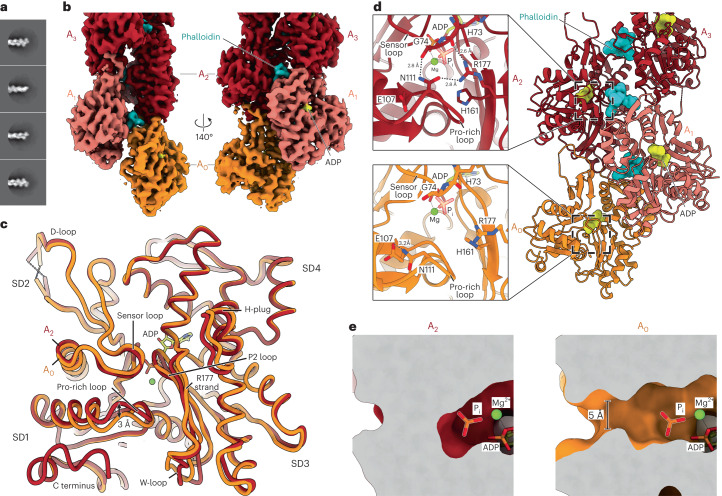
Cryo-EM structure of the phalloidin-bound barbed end of F-actin. **a**, Exemplary two-dimensional (2D) class averages of the barbed-end particles. The particle boxes are 465 × 465 Å^2^. Additional 2D class averages are depicted in Extended Data Figure 1b. **b**, Cryo-EM density map of the barbed end at 3.6-Å resolution, shown in two orientations. Actin subunits with complete inter-subunit contacts (A_2_ and above) are colored dark red, the penultimate subunit (A_1_) is colored salmon, and the ultimate subunit (A_0_) is depicted in orange. Phalloidin (cyan) and ADP (yellow) are annotated. **c**, Superimposition of the A_2_ and A_0_ subunits at the barbed end. Actin subdomains (SD1–SD4) and the regions that are different between the two subunits are annotated. P2 loop, residues 154–161. R177 strand, residues 176–178. **d**, Right, molecular model of the F-actin barbed end. The images on the left depict a zoom-in on the R177–N111 backdoor as seen from the F-actin exterior. Amino acids that form the backdoor are annotated. Although the subunits adopt the ADP state, P_i_ from PDB 8A2S (F-actin in the Mg^2+^-ADP-P_i_ state) is fitted and shown semi-transparently to emphasize the P_i_-binding site. **e**, A slice through the structures of the A_2_ and A_0_ subunits near the nucleotide-binding site, shown in surface representation. Similar to **d**, P_i_ from PDB 8A2S is shown to emphasize the P_i_-binding site.

**Extended Data Fig. 1:**
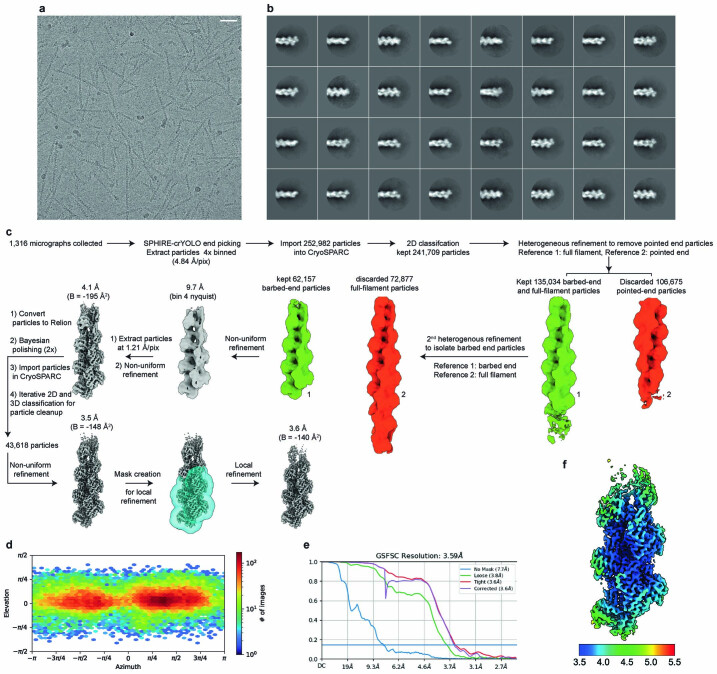
Cryo-EM image-processing of the F-actin barbed end. **a,** Micrograph depicting short β/γ-actin filaments frozen in vitreous ice, at a defocus of −2.2 µm. The shown micrograph is an example image from a total dataset of 1,316 micrographs. The scale bar is 400 Å. **b**, Exemplary 2D-class averages of the F-actin barbed end reconstruction, generated by RELION. The box size is 465 $\times$ 465 Å^2^. **c,** Image processing strategy that was employed to determine the β/γ-actin barbed end structure. All maps are shown in the same orientation. **d,** Angular distribution of the barbed end particles used to reconstruct the final cryo-EM map, generated by CryoSPARC. **e,** Fourier-shell correlation plots for the barbed structure of gold-standard refined half-maps, computed by CryoSPARC. The FSC = 0.143 threshold is annotated. **f,** Local-resolution estimation of the barbed end reconstruction, computed through RELION.

To elucidate how the conformation of the barbed end of F-actin allows for much faster P_i_-release kinetics during filament depolymerization, we first sought to solve the structure of the barbed end by cryo-EM. This required the generation of short filaments (<150 nm) so that we could pick enough filament ends per micrograph to determine the structure of the barbed end in high resolution. In vitro, actin polymerization generally results in long filaments (>500 nm) because filament growth is kinetically favored over nucleation. We therefore optimized a workflow for generating short filaments that featured DNase I, a G-actin-binding protein^34^ that depolymerizes F-actin under physiological conditions. However, in the presence of the toxin phalloidin, DNase I does not disassemble F-actin and acts effectively as a pointed-end capper^35^ that prevents filament reannealing. Accordingly, to generate short filaments, we mixed DNase I–G-actin complex with free G-actin and the FH2 domain of formin mDia1 (mDia1_FH2_, which acts as an actin nucleator) in low-ionic-strength buffer, and added KCl to induce polymerization. Shortly after, we stabilized the formed filaments with phalloidin and then separated F-actin from unpolymerized actin and mDia1_FH2_ using size-exclusion chromatography (SEC). Through this approach, we reproducibly formed short (~50–150 nm length) filaments and finally obtained a cryo-EM structure of the barbed end at 3.6-Å resolution (Fig. 1a,b, Extended Data Fig. 1, Table 1 and Supplementary Video 1). Importantly, we did not find any evidence of an ABP that remained bound to the barbed end, indicating that our structure was the undecorated barbed end of F-actin.

**Table 1:** Cryo-EM data collection, refinement and validation statistics

dataset	β/γ-actin barbed end	β-actin-R183W	β-actin-N111S
	EMD-16887, PDB 8OI6	EMD-16888, PDB 8OI8	EMD-16889, PDB 8OID
**Data collection and processing**			
Magnification	×120,000	×130,000	×130,000
Voltage (kV)	200	300	300
Electron exposure (e^–^/Å^2^)	56	70	69
Defocus range (μm)	−1.2 to −2.7	−0.7 to −2.0	−0.7 to −2.0
Pixel size (Å)	1.21	0.695	0.695
Symmetry imposed	*C*_1_	*C*_1_	*C*_1_
Initial particle images (no.)	252,982	1,569,882	2,001,281
Final particle images (no.)	43,618	1,286,604	1,756,928
Map resolution (Å)			
0.143 FSC threshold	3.59	2.28	2.30
Map resolution range (Å)	3.5–5.5	2.2–3.7	2.2–3.9
Measured helical symmetry^a^			
Helical rise (Å)	27.47	27.58 ± 0.08	27.58 ± 0.05
Helical twist (^o^)	−165.7	−166.5 ± 0.1	−166.5 ± 0.3
**Refinement**			
Initial model used (PDB code)	8A2T	8OID	8A2T
Model resolution (Å)			
0.5 FSC threshold	3.79	2.30	2.30
Map sharpening *B* factor (Å^2^)	−140	−55	−60
Model composition			
Non-hydrogen atoms	11,573	14,895	15,206
Protein residues	1,464	1,815	1,850
Waters	0	585	616
Ligands	8	10	10
*B* factors (Å^2^)			
Protein	76.52	32.06	31.10
Waters	−	27.36	27.47
Ligand	69.41	24.35	21.49
R.m.s. deviations			
Bond lengths (Å)	0.002	0.004	0.004
Bond angles (°)	0.589	0.568	0.597
Validation			
EM-ringer score	1.83	5.56	5.49
MolProbity score	1.42	1.13	1.45
Clashscore	7.67	3.42	4.07
Poor rotamers (%)	0.24	0.65	0.96
Ramachandran plot			
Favored (%)	98.10	98.60	96.16
Allowed (%)	1.83	1.40	3.84
Disallowed (%)	0.07	0	0

^a^For the β/γ-actin barbed-end structure, the helical rise and twist between the ultimate (A_0_) and penultimate (A_1_) subunit are reported. For the filament structures, the helical parameters were estimated from the atomic model of five consecutive subunits independently fitted to the map, as described previously^8^.

**Extended Data Fig. 2:**
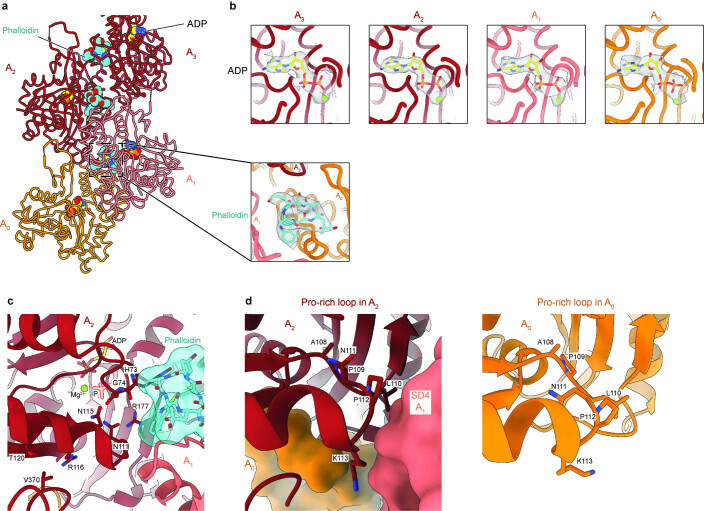
Small-molecule binding sites and Pro-rich loop arrangement in the F-actin barbed end structure. **a**, Simplified cartoon representation of the barbed end structure. Actin subunits are annotated. ADP and phalloidin are shown as spheres with carbon atoms colored cyan and yellow, respectively. **b,** Cryo-EM densities of the ADP and phalloidin binding sites with fitted models. F-actin is shown as simplified cartoon. **c,** Zoom of the phalloidin-binding site near the R177-N111 backdoor in actin subunit A_2_. Phalloidin is shown in stick representation with a semi-transparent surface. Residues of the proposed backdoor (R177, N111, H73, G74) are annotated, as well as other residues (N115/R116, T120/V370) that may represent other P_i_ escape sites. Phalloidin would only interfere with disruption of the classical backdoor. **d,** Arrangement of the Pro-rich loop in subunits A_2_ (left panel) and A_0_ (right panel) in the barbed end structure. In subunit A_2_, the Pro-rich loop conformation is stabilized by SD4 of subunit A_1_. Conversely, the Pro-rich loop of subunit A_0_ does not interact with other subunits.

**Extended Data Fig. 3:**
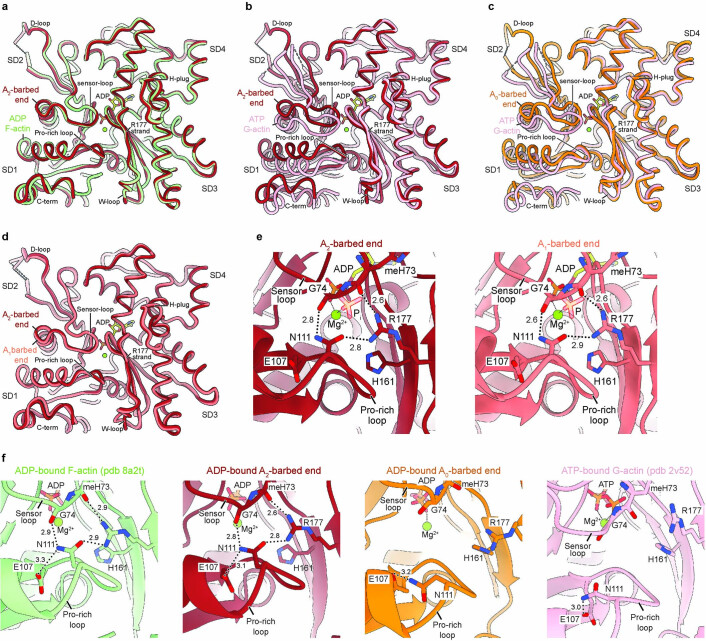
Alignment of barbed-end subunit structures with previously determined actin structures. **a,** Superimposition of the A_2_ subunit of the barbed end structure (colored dark-red) with the structure of ADP-bound filamentous α-actin (pdb 8a2t, colored green). **b,** Superimposition of the A_2_ subunit of the barbed end structure with the structure of ATP-bound monomeric α-actin (pdb 2v52, colored pink). **c**, Superimposition of the A_0_ subunit of the barbed end structure (colored orange) with the structure of ATP-bound monomeric α-actin (pdb 2v52). **d,** Superimposition of the A_2_ (colored dark-red) and A_1_ (colored salmon) subunits of the barbed end structure. The only observed change in penultimate subunit A_1_ is a small rearrangement of the W-loop. This change in the W-loop is expected, because this region would interact with the D-loop of the missing subunit. **e,** Zoom of the R177-N111S backdoor as seen from the F-actin exterior for the A_2_ and A_1_ subunits of the barbed end structure. Amino acids that form the backdoor are annotated. Phalloidin is hidden for clarity. Although the subunits adopt the ADP state, P_i_ from pdb 8a2s (F-actin in the Mg^2+^-ADP-P_i_ state) is shown semi-transparently to emphasize the P_i_-binding site. **f**, Architecture of the P_i_-release backdoor in structures of ADP-bound F-actin (left), the ADP-bound A_2_ subunit at the F-actin barbed end (middle-left), the ADP-bound A_0_ subunit at the F-actin barbed end (middle-right) and ATP-bound G-actin (right).

The structure reveals the hallmark actin filament architecture of a double-stranded helix with a right-handed twist (Fig. 1b,d). All actin subunits in our reconstruction adopt the aged ADP-nucleotide state (Extended Data Fig. 2a,b), indicating that we captured the barbed-end structure in a state that resembles a depolymerizing filament. This is in line with our experimental setup, in which the filaments were separated from actin monomers after polymerization. In the structure, the arrangements of F-actin subunits that make all available inter-subunit contacts within the filament (A_2_ and above), as well as the arrangement of the penultimate subunit A_1_, are essentially the same as in previously determined F-actin structures (Extended Data Fig. 3, Cα r.m.s. deviation (r.m.s.d.) < 0.5 Å with PDB 8A2T) (ref. ^8^). Accordingly, in subunits A_1_ and above, the predicted R177–N111 backdoor is closed and the P_i_-binding site is shielded from the filament exterior (Fig. 1d and Extended Data Fig. 3e). Although the overall arrangement of the ultimate (A_0_) subunit is also similar (Cα r.m.s.d. of 0.9 Å between subunits A_0_ and A_2_) and remains flattened (Extended Data Fig. 3c), we observed several differences when we compared it with the internal subunit A_2_, including small rearrangements in the W-loop (residues 165–172) and in the hydrophobic plug region (residues 264–273), as well as a disordered F-actin carboxy terminus (residues 363–375) (Fig. 1c). However, the most striking rearrangement is the downward displacement of the Pro-rich loop (residues 107–112) by ~3 Å (Fig. 1c), which—unlike in internal actin subunits—is not stabilized by subdomain 4 (SD4) of an adjacent subunit (Extended Data Fig. 2d). Hence, N111 loses its hydrogen bonds with G74 in the sensor loop (residues 70–77) and SD3-residue R177 and instead interacts with E107, within its own local loop (Fig. 1d and Extended Data Fig. 3f). Interestingly, this E107-N111 interaction is commonly observed in G-actin structures (Extended Data Fig. 3f). Strikingly, residue H161, which flips rotameric position during the G- to F-actin transition and is important for ATP hydrolysis in F-actin^7,8^, also adopts its G-actin-like position in the A_0_ subunit and points towards R177. As a result, R177 can no longer interact with the sensor loop (Fig. 1d and Extended Data Fig. 3f). In conclusion, the hydrogen bonding network formed by R177, N111, H73, and G74 is fully abolished, which opens a hole of ~5 Å in diameter in the structure that connects the internal nucleotide-binding site to the filament exterior (Fig. 1e and Supplementary Video 1). This defines the predicted P_i_ release backdoor ^29^ as open. Thus, our structure provides evidence that, under depolymerization conditions, P_i_ can dissociate from the nucleotide-binding site at the barbed end without large protein rearrangements, thereby revealing the structural basis for why the ultimate barbed end subunit of actin releases P_i_ at rates that are orders-of-magnitude faster than those from subunits within the filament core.

### Two plausible P_i_-egress routes from the F-actin core

**Fig. 2:**
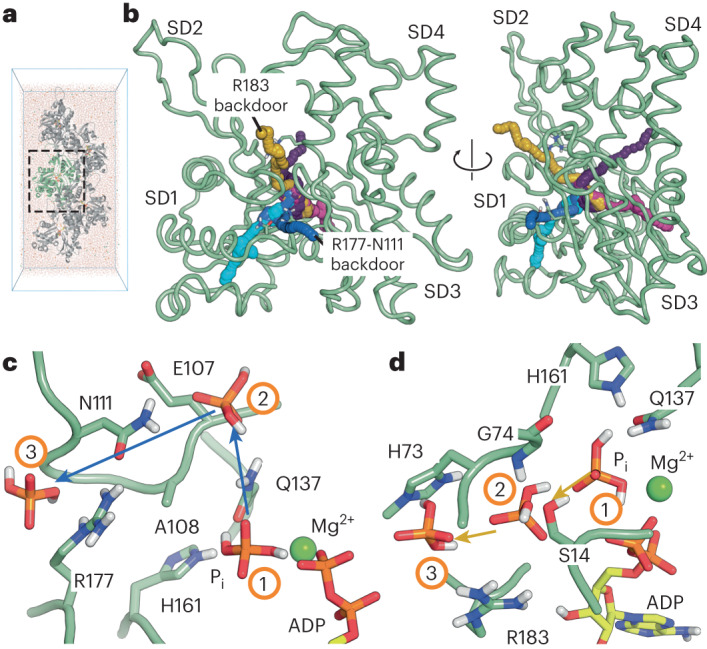
Enhanced sampling simulations reveal several P_i_-release paths from the F-actin core. **a**, Simulation box containing an explicitly solvated actin pentamer. The core actin subunit is shown in pale green. **b**, Typical P_i_-egress pathways from the actin core, obtained by enhanced sampling. Actin subdomains are annotated. The two plausible egress paths that were analyzed further are the putative R183 backdoor (gold) and R177-N111 backdoor (blue). Implausible P_i_ release pathways are colored purple and magenta and are further shown in Extended Data Figure 4. The F-actin structure fluctuated during the simulations but is shown in a single, representative conformation for clarity. **c**,**d**, Close-up views of the plausible P_i_-egress paths. Time-ordered representative P_i_ positions (1–3) from enhanced sampling trajectories are shown and connected by arrows indicating the direction of P_i_ movement. Similar to **b**, the F-actin structure is shown in a single, representative conformation for clarity. Close-up views are depicted for the potential R177-N111 backdoor path (**c**) and for the potential R183 backdoor path (**d**).

**Extended Data Fig. 4:**
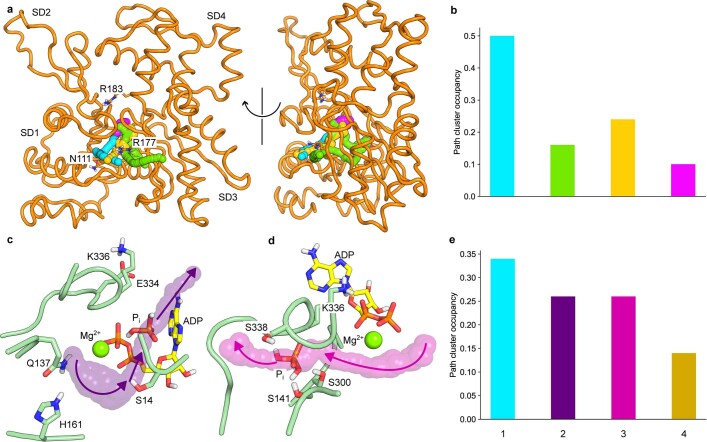
Enhanced sampling simulations of P_i_ release from the F-actin barbed end and core. **a,** Representative egress paths from the barbed end show a clear preference for egress through the open R177-N111 backdoor. The F-actin structure fluctuated during the simulations but is shown in a single, representative conformation for clarity. **b**, Path cluster occupancies in barbed end simulations with the same color code as in panel **a**. **c**, A representative path of P_i_ egress through pathway 2 (purple) in F-actin core simulations. The simulation revealed a highly bent ADP conformer that allowed P_i_ to escape. Such a drastic rearrangement of the nucleotide binding pocket is physically implausible. In addition, this egress path would not be directly affected by phalloidin and jasplakinolide binding. Hence, this pathway was not considered for further experimental validation. **d,** A representative path of P_i_ egress through pathway 3 (magenta) in F-actin core simulations. This escape would also not be directly affected by phalloidin and jasplakinolide binding. Hence, it was not considered for further experimental validation. Similar to panel a, the F-actin structure is shown in a single, representative conformation for clarity. **e,** Path cluster occupancies in actin core simulations with the same color code as in Fig. 2. Pathways 1 and 4 are further discussed in the main text.

The barbed-end structure revealed a P_i_-release pathway similar to the path that has been predicted in previous MD simulation studies^29^. Accordingly, when we performed MD simulations on P_i_ release from barbed-end subunit A_0_, we observed that virtually all P_i_-escape events occurred through the open R177-N111 backdoor (Extended Data Fig. 4a,b). However, we reasoned that other P_i_-exit routes may exist for F-actin core subunits, which make all available inter-subunit contacts and release P_i_ at much slower rates. We therefore set out to develop an MD protocol to investigate the P_i_-release mechanism from the F-actin filament core, using our recently reported ~2.2-Å structure of F-actin in the Mg^2+^-ADP-P_i_ state^8^ as a high-quality starting model (Fig. 2a). Because P_i_ release from the F-actin core is a slow, stochastic event with half-times >100 s, the diversity of possible P_i_ escape paths cannot be studied using conventional MD simulations. Instead, we developed an enhanced-sampling simulation protocol based on metadynamics by applying a history-dependent repulsive potential to the P_i_ Cartesian coordinates to progressively drive P_i_ out of the nucleotide-binding site^36^, without favoring any egress route a priori (see Methods). This protocol enables simulation of candidate P_i_-egress pathways in about 10 ns.

Using this approach, we collected dozens of P_i_-release events from the F-actin core and identified sterically accessible egress pathways (Fig. 2b). We then analyzed the diverse-sampled pathways for physical plausibility and first dismissed all pathways that entailed unrealistic distortions of F-actin or the nucleotide (Extended Data Fig. 4c–e). Second, we assumed that P_i_ exits the F-actin interior near the binding sites of phalloidin and jasplakinolide, because both toxins strongly inhibit P_i_ release^37–39^. This analysis resulted in two remaining egress pathways that were physically plausible. In the first, a side chain movement of Q137 allows P_i_ to move into a hydrophilic pocket between E107 and H161, where it interacts with residues in the Pro-rich loop. From there, P_i_ escapes either by disrupting the R177-N111 hydrogen bond (leading to an open backdoor, similar to the conformation adopted by the barbed end) (Fig. 2c and Supplementary Video 2), by leaving close to residues N115 and R116, or by exiting near residues T120 and V370. Of note, phalloidin and jasplakinolide stabilize the R177-N111 interaction (Extended Data Fig. 2c), but would not interfere with P_i_ egress near N115-R116 or T120-V370, suggesting that the exit path that requires the disruption of the R177-N111 interaction is the most probable. In the second plausible pathway, P_i_ first breaks the hydrogen bond between S14 and G74 to enter a pocket between H73 and R183. Then, P_i_ escapes to the intra-filament space upon breaking the strong electrostatic interaction with R183 (Fig. 2d). Interestingly, the disruption of the S14-G74 hydrogen bond has previously been proposed to play a role in P_i_ release, albeit through a different mechanism^33^. Furthermore, phalloidin and jasplakinolide would prevent the opening of the H73-R183 pocket. Thus, in addition to the predicted route, there is another physically realistic egress pathway for P_i_ from the F-actin core.

### Actin filaments with the N111S substitution release P_i_ rapidly

To experimentally probe the two possible P_i_-release pathways from the F-actin core, we mutated key residues in β-actin that pose a barrier for P_i_ release in each pathway. For the first pathway, we introduced the p.N111S substitution to potentially disrupt the hydrogen bonding network of the R177-N111 backdoor. For the second pathway, we aimed to destabilize the putative R183-mediated backdoor using p.R183W and p.R183G mutations. Importantly, all actin mutants studied here are associated with human diseases; p.R183W was identified in β-actin from patients with deafness, juvenile-onset dystonia or development malfunctions^40,41^, whereas p.R183G and p.N111S have been found in α-actin of patients with nemaline myopathy^42,43^, highlighting the relevance of these actin variants.

**Fig. 3:**
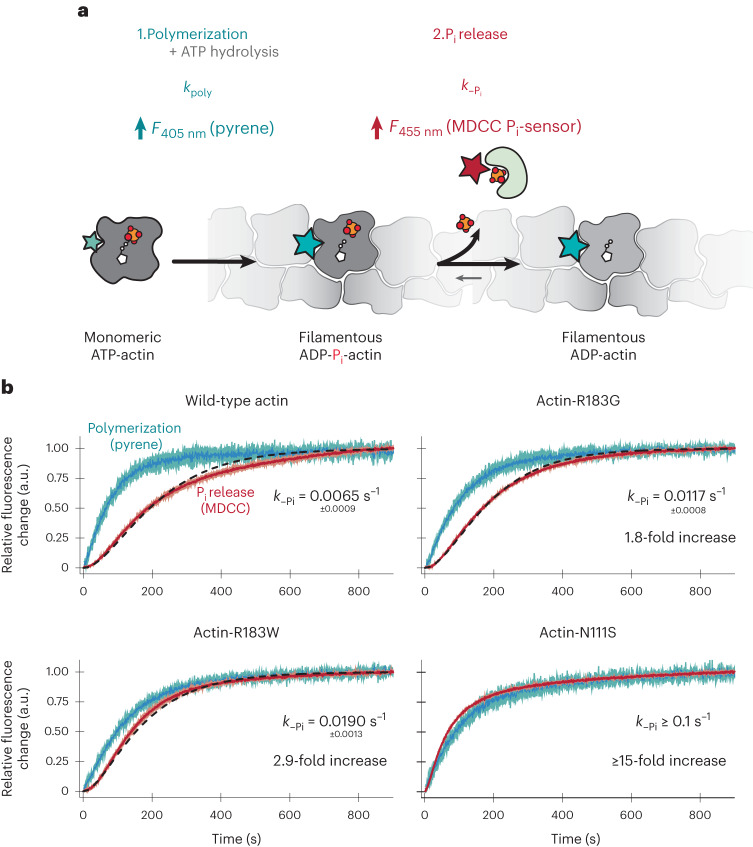
Biochemical characterization of P_i_ release from actin filaments. **a**, Scheme of the synchronous measurement of actin polymerization and subsequent P_i_ release as measured by the fluorescence intensity increase of pyrene (cyan) and MDCC-PBP (red), respectively. *k*_poly_, apparent rate of actin polymerization; *k*_–Pi_, rate constant for phosphate release. F_405 nm_ and F_455 nm_ refer to the measured increase in fluorescent intensity at the specified emission wavelength. **b**, Timecourses of the normalized fluorescence intensities of 10 µM actin (either wild-type or mutants as indicated) containing 1.5% wild-type, pyrene α-actin (cyan), and 30 µM MDCC-PBP (red) seeded with 160 nM spectrin-actin seeds after initiation of polymerization (*t* = 0 s). Dark colors indicate the average of three independent experiments, whereas the lighter colored areas indicate the s.d. The black dashed lines correspond to fits of the phosphate release data to a kinetic model (see Methods). Rates of P_i_ release and relative rate enhancement over wild-type actin as determined either from fits to a kinetic model (wild-type, actin-R183G and actin-R183W) or by estimation from kinetic simulations (actin-N111S, see Methods and Extended Data Fig. 5c,d) are depicted in each graph. Source data

**Extended Data Fig. 5:**
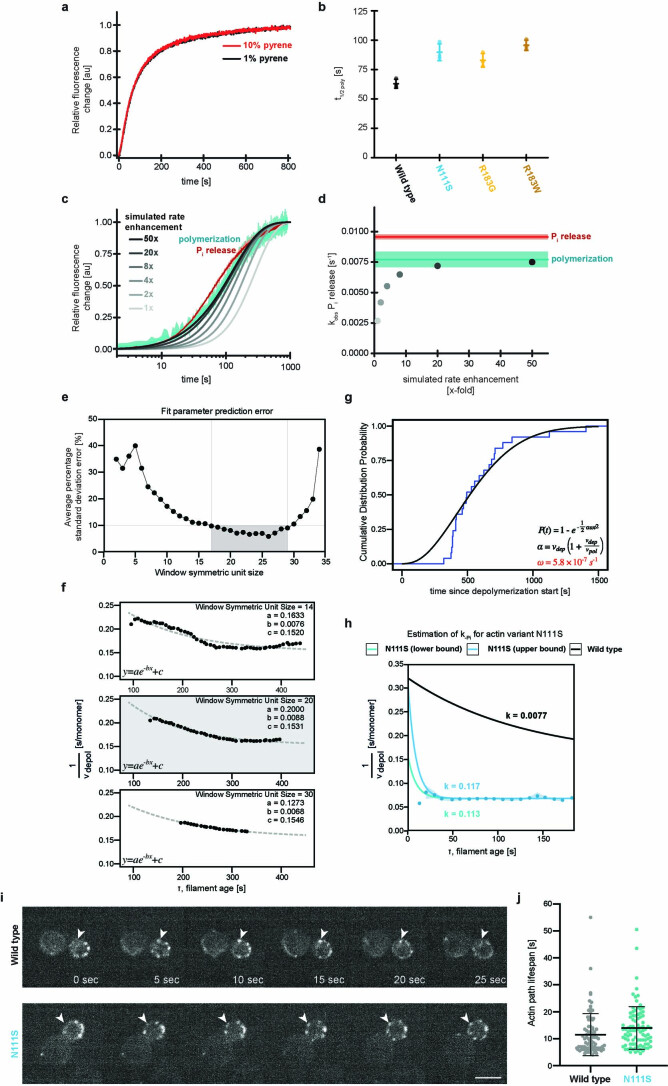
Kinetic analysis of P_i_ release from actin filaments and dynamics of actin variants in yeast. **a,** Time courses of the normalized fluorescence intensity of 30 µM MDCC-PBP (P_i_ sensor) from 10 µM N111S actin containing either 1% (black) or 10% (red) wild-type, pyrene α-actin, seeded with 100 nM spectrin-actin seeds after initiation of polymerization (t = 0s). **b**, Characteristic half-times of polymerization for indicated actin variants as determined from mono-exponential fits to the observed polymerization time-courses (Fig. 3b). Dots are values from individual experiments, whereas lines represent the mean from three experiments. Error bars are SD. **c,** Semi-logarithmic plot of simulated P_i_ release reaction kinetics (gray to black) depending on the enhancement of P_i_ release rate constant (x-fold over wild-type actin as indicated) compared to the observed time courses of polymerization (cyan) and of P_i_ release (red) for N111S actin. **d,** Apparent rates of P_i_ release, obtained from mono-exponential fits of the simulated data shown in c as a function of the enhancement of P_i_ release rate constant (x-fold over wild-type actin as indicated). The cyan and red areas indicate the observed apparent rates of polymerization (cyan) and P_i_ release (red) with the center of the error bands (dark colors) being the average from three experiments and the error bands (light colors) representing SD. Note that an enhancement of the P_i_ release rate constant by at least 15-fold is required for the apparent rate of P_i_ release to fall within the error margin of the observed polymerization rate. **e,** Effect of varying window sizes on the average prediction error of the three fit parameters from the kinetic model. The average prediction error is large (>10%) for small and large window sizes with a shallow optimum in between (grey shaded region, size 17 to 29). At smaller window sizes the prediction error is large as a result of a higher variance in calculated velocities and for larger window sizes the prediction error increases with decrease in the number of data points available for fitting. **f**, Exemplary fits (grey dashed lines) calculated from velocities (black points) obtained with different window sizes from within (shaded grey, size 20) and flanking (size 14 and 30) the optimum. The exponential decay function used for these fits and their fit parameters are provided in the bottom-left and top-right parts of each sub-plot respectively. **g**, Cumulative distribution probability of pauses as a function of time from the onset of depolymerization. Blue line indicates the fraction of tracked filaments that have paused at any given time and the black line is obtained by fitting an analytic model of pause probability (described previously in ref. ^70^) as a function of time. The model is described by the equations in the bottom-right corner of the plot. From this fit we obtained for the fit parameter $\omega$ (protomer transition rate) a value of 5.8 × 10^−7^ s^−1^. **h**, Estimation of lower bounds for the phosphate release rate of N111S mutant. Rates were calculated by fitting exponential decay functions to the observed depolymerization velocity data, so that the observed velocities converged to the mutant’s v_depol,ADP_ and the v_depol,ADP-Pi_ rate was either assumed to be equal (high estimate, blue) or twice that of the wild type (low estimate, turquoise). The latter assumption was motivated by the observation that the v_depol,ADP_ rate of N111S mutant was about 2.2 times that of wild-type actin (Fig. 5d). **i**, Time-lapse series of confocal microscopy images showing Lifeact-mCherry fluorescence every 5 seconds for actin path detection in yeast cells expressing either wild-type or N111S-actin. The white arrow highlights an exemplary dynamic actin path. The scale bar is 4 µm. The shown images of yeast cells are example images from a total of eight cells per strain. Four independent colonies per strain were imaged. The full experiment was performed in duplicate, obtaining similar results. **j,** Plot of endocytic actin patch lifespans per strain. Each color dot represents one patch. The average lifespans are 12 s ± 7 s (wild-type actin) and 14 s ± 7 s (N111S-actin). Error bars correspond to mean ± standard deviation. Sample size corresponds to n = 86 patches for WT and n = 89 patches for N111S, from a total of eight cells per strain from four independent colonies. There are no large differences in actin-patch lifespans between the two yeast strains. Source data

We developed a fluorescence-based assay to synchronously monitor seeded actin polymerization using pyrene fluorescence and subsequent P_i_ release via a fluorescent phosphate sensor in the same experiment (Fig. 3a). This allowed us to determine the respective reaction rates by fitting the data to a kinetic model (see Methods). Because we performed the experiments at actin concentrations (10 μM) that drive rapid filament growth and seeded the reaction with spectrin-actin seeds to circumvent slow nucleation, we effectively monitored P_i_ release from core subunits and not from the barbed end. Wild-type β-actin had a slow P_i_ release rate of 0.0065 s^−1^ (*τ* ≈107 s) (Fig. 3b), which falls within the range of previously reported values for rabbit skeletal α-actin^10,21,25^, indicating that slow P_i_ release after polymerization and ATP hydrolysis is a conserved feature among mammalian actin isoforms. Actin-R183G and actin-R183W released P_i_ at slightly increased rates of, respectively, 0.0117 s^−1^ and 0.0190 s^−1^, corresponding to a 1.8- and 2.9-fold increase compared with wild-type β-actin (Fig. 3b). Strikingly, actin-N111S exhibited ultrafast P_i_ release kinetics without appreciable delay after polymerization. In fact, the reaction timecourses of P_i_ release slightly outpaced the observed polymerization kinetics (Fig. 3b), making it impossible to determine an exact P_i_ release rate for actin-N111S. However, when estimating the release rate conservatively (see Methods and Extended Data Fig. 5c,d), actin-N111S releases P_i_ at a rate of ≥0.1 s^−1^, which is ≥15-fold faster than the rate of wild-type actin. Thus, the R183 mutants release P_i_ somewhat faster, but still display the characteristic delay between polymerization and P_i_ release; by contrast, actin-N111S appears to release P_i_ without appreciable delay.

### Structural basis for the fast P_i_-release rates of actin-N111S

**Fig. 4:**
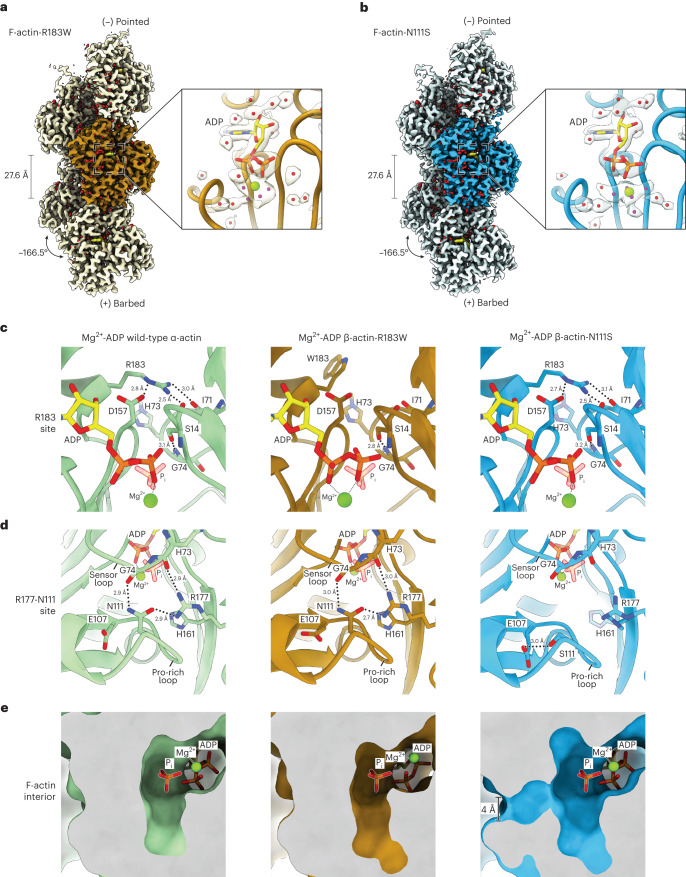
High-resolution cryo-EM structures of F-actin-R183W and F-actin-N111S. **a**,**b**, Sharpened cryo-EM density maps of F-actin-R183W (**a**) and F-actin-N111S (**b**) in the Mg^2+^-ADP nucleotide state. Five filament subunits are shown. For the R183 mutant, the central subunit is colored gold; the central subunit of the N111S mutant is colored blue. The helical rise and twist are annotated. Densities corresponding to water molecules are shown in red. For each mutant, a zoom-in of the nucleotide-binding pocket with cryo-EM densities for the nucleotide (depicted in yellow), the Mg^2+^ ion (green), and water molecules (red) is shown. Water molecules that directly coordinate the Mg^2+^ ion are colored magenta. The polar ends of the actin filament, (−) pointed and (+) barbed, are annotated. **c**–**e**, Structural comparison between filamentous wild-type α-actin (PDB 82AT, left, green), β-actin-R183W (middle, gold), and β-actin-N111S (right, blue) in the Mg^2+^-ADP state. The panels depict the amino acid environment near the location of residue 183 (**c**), the amino acid environment near residues 177 and 111 (**d**), and a slice through the F-actin interior near the P_i_-binding site (**e**). In **c** and **d**, F-actin is shown as a cartoon, and amino acids are shown as sticks and are annotated. In **e**, F-actin is depicted in surface representation. Water molecules are omitted from **c**–**e**. Although the depicted structures adopt the ADP state, P_i_ from PDB 8A2S (F-actin in the Mg^2+^-ADP-P_i_ state) is fitted and shown semi-transparently to emphasize the P_i_-binding site.

**Extended Data Fig. 6:**
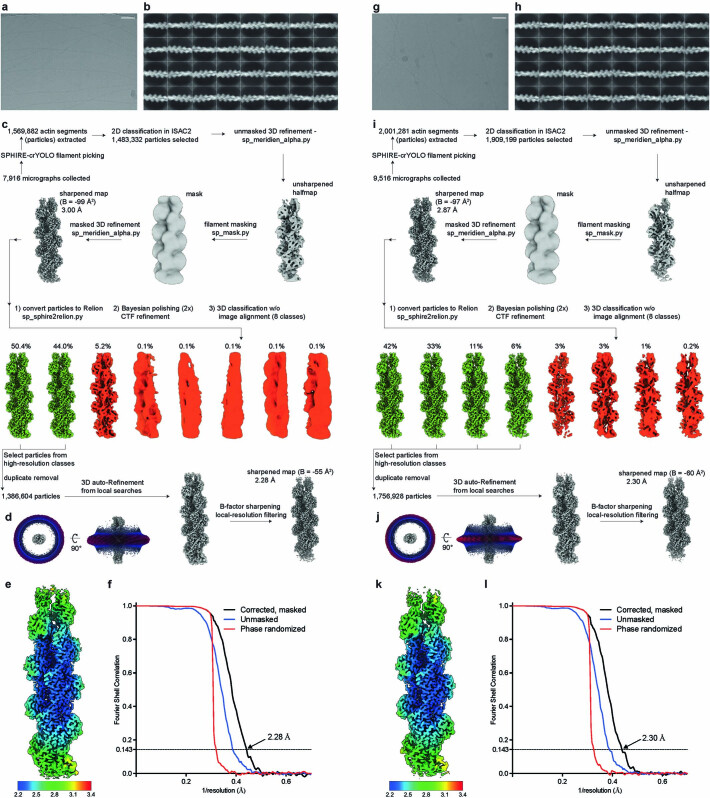
Processing of the F-actin-R183W and F-actin-N111S datasets. **a, g,** Micrographs depicting R183W (**a**) and N111S (**g**) variants of F-actin frozen in vitreous ice, at, respectively, defocus values of −2.3 µm and −1.9 µm. The shown micrographs are example images from total datasets of 7,916 (R183W) and 9,516 (N111S) micrographs. The scale bars are 400 Å. **b, h,** Exemplary 2D-class averages of the R183W- (**b**) and N111S-F-actin (**h**) particles, computed through RELION. The box size is 267 ${\rm{\times }}$ 267 Å^2^. **c**, **i,** Image processing strategies that were employed to determine the actin-R183W (**c**) and actin-N111S (**i**) structures. All maps are shown in the same orientation. **d, j**, Angular distribution of the particles used to reconstruct the final cryo-EM maps of F-actin-R183W (**d**) and F-actin-N111S (**j**), shown along the filament axis (left) and orthogonal to the filament axis (right). **e, k,** Local-resolution estimations of the R183W- (**e**) and N111S-F-actin (**k**) density maps, calculated by RELION. The bar depicts local resolution in Å. **f**, **l**, Fourier-shell correlation plots for gold-standard refined masked (black), unmasked (blue) and high-resolution phase randomized (red) half-maps of F-actin-R183W (**f**) and F-actin-N111S particles (**l**). The FSC = 0.143 threshold is shown as a dashed line.

**Extended Data Fig. 7:**
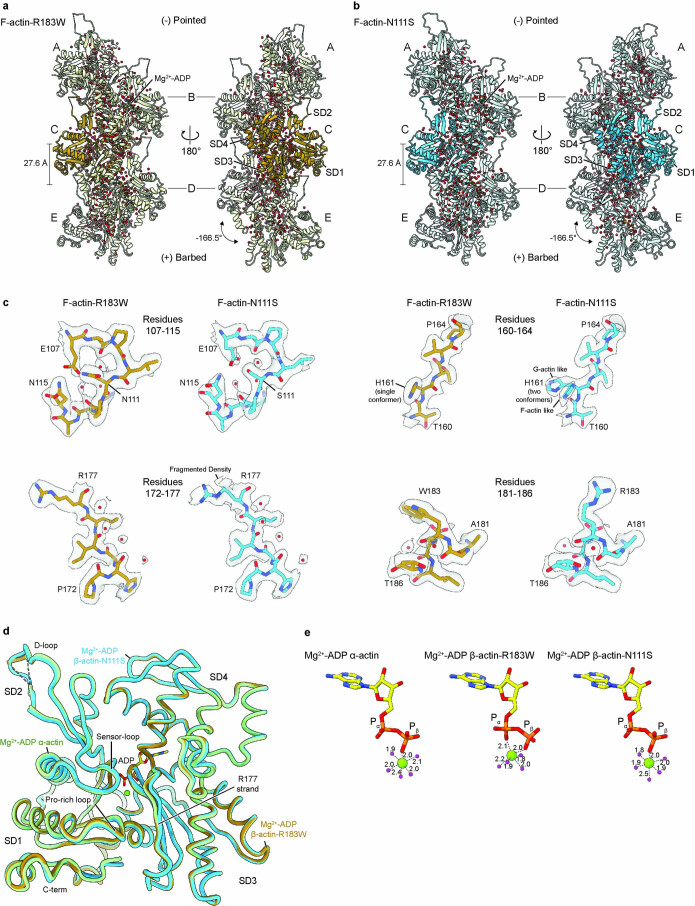
High-resolution structures of β-actin variants R183W and N111S. **a, b,** Structures of F-actin-R183W **(a)** and F-actin-N111S **(b)** shown as cartoon orthogonal to the filament axis. The central subunits are colored gold and blue, respectively. Water molecules modeled in both structures are depicted as red spheres. The pointed and barbed end directions are annotated, as well as the helical rise and twist of both mutant structures. **c**, Cryo-EM densities and fitted atomic models for selected regions of both reconstructions. Specifically, amino-acid environments near the mutated residues are shown to highlight differences between both structures. **d**, Alignment of single subunits of filamentous wild-type α-actin (pdb 8a2t, colored green), R183W-β-actin (gold) and N111S-β-actin (blue) in the Mg^2+^-ADP state. The F-actin subdomains and regions important for P_i_ release are annotated. **e,** Nucleotide arrangement in high-resolution Mg^2+^-ADP-bound F-actin structures of wild-type α-actin (pdb 8a2t, left), β-actin-R183W (middle) and β-actin-N111S (right). The arrangements in wild-type α-actin and β-actin-N111S are similar, whereas the Mg^2+^ ion adopts a different position in the β-actin-R183W structure.

To structurally understand the differences in P_i_-release rates between the R183W and N111S mutants, we determined the filament-core structures of these variants in the Mg^2+^-ADP-bound state at ~2.3-Å by cryo-EM (Fig. 4a,b, Table 1 and Extended Data Figs. 6 and 7a,b). We modeled hundreds of water molecules bound to F-actin in both structures, as well as the exact rotameric positions of many amino acid side chains (Extended Data Fig. 7a–c). Globally, we observed no major differences between the two β-actin mutants and Mg^2+^-ADP-bound α-actin filaments (PDB 8A2T, Cα r.m.s.d. < 0.7 Å) (Extended Data Fig. 7d) but, importantly, we identified small but impactful rearrangements.

We first examined the atomic arrangement near the mutated residues. In wild-type F-actin structures, the R183 side chain interacts with D157 and the carboxyl moieties of S14 and I71 (Fig. 4c). As expected for F-actin-R183W, these interactions are abolished and the W183 side chain points away from the mentioned residues (Fig. 4c). Nevertheless, the S14-G74 hydrogen bond remains intact in the mutant, and we did not observe an open cavity near W183 through which P_i_ could escape (Fig. 4c); the R177-N111 backdoor is also intact (Fig. 4d,e).

The N111S substitution induces a more drastic conformational change in F-actin; residue S111 is too short to interact with R177 and G74. Instead, S111 forms a hydrogen bond with E107, the backbone of A108, and a water molecule within its local environment in the Pro-rich loop (Fig. 4d and Extended Data Fig. 7c). Additionally, the density for the side chain of R177 is fragmented (Extended Data Fig. 7c), indicating that the residue is more flexible than in wild-type F-actin structures. As a result, the interaction between the sensor loop and Pro-rich loop is disrupted and a ~4-Å-diameter hole is observed, yielding an open backdoor (Fig. 4d,e). Hence, the conformation of F-actin-N111S subunits is reminiscent of the arrangement of the ultimate subunit of the barbed-end structure (Fig. 1d). Accordingly, we identified that H161 adopts a mixture of rotameric states and that it partially adopts a G-actin-like conformation (Fig. 4d and Extended Data Fig. 7c). This observation suggests that repositioning of the H161 side chain is a key feature of backdoor opening. Conversely, the S14-G74 interaction is not affected in F-actin-N111S (Fig. 4c), indicating that disruption of this interaction is not required to open the backdoor, as has previously been proposed^33^. Thus, although the backbone atom shifts in actin-N111S are minor when compared with those in wild-type actin (< 1 Å), side chain rearrangements result in a broken hydrogen bonding network and an open backdoor (Fig. 4d,e, Extended Data Fig. 7c and Supplementary Video 3), defining the structural basis for the ultrafast P_i_-release kinetics of F-actin-N111S.

**Extended Data Fig. 8:**
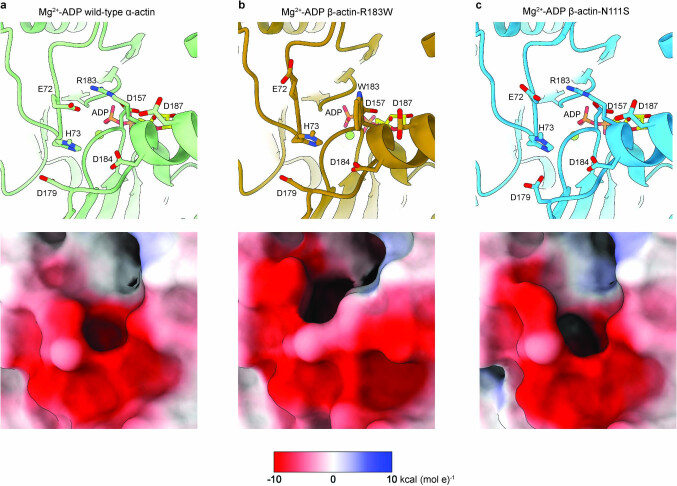
Acidic amino-acid environment around residue 183. **a–c,** Arrangement near residue 183, as show from the filament exterior for filamentous wild-type α-actin **(a)** (pdb 8a2t, colored green), β-actin-R183W (gold) **(b)** and β-actin-N111S (blue) **(c)** in the Mg^2+^-ADP state. The top image depicts F-actin as cartoon and charged amino acids near residue-183 as cartoon and sticks. In the lower panel, F-actin is shown as surface, which is colored by electrostatic Coulomb potential ranging from −10 kcal (mol e)^−1^ (red) to +10 kcal (mol e)^−1^ (blue). The surface is more negatively charged in the F-actin-R183W structure.

We then inspected the conformation of ADP and the associated Mg^2+^ ion in both mutant structures. In the F-actin-N111S structure, the nucleotide arrangement is essentially the same as that in Mg^2+^-ADP wild-type α-actin; the Mg^2+^ ion resides beneath the β-phosphate of ADP and interacts with five water molecules (Fig. 4b, Extended Data Fig. 7e and Supplementary Video 3). Interestingly, in F-actin-R183W, Mg^2+^ changes position so that it is directly coordinated by both α- and β-phosphates, as well as by four water molecules (Fig. 4a and Extended Data Fig. 7e). Although R183 does not directly interact with ADP in wild-type F-actin, it resides in close proximity to the nucleotide and, importantly, it is positioned in a negatively charged cluster of acidic residues (Extended Data Fig. 8). Hence, F-actin-R183W has a more negatively charged nucleotide-binding site (Extended Data Fig. 8), which may explain why the positively charged Mg^2+^ repositions in the mutant structure to compensate for the charge imbalance. Thus, F-actin-R183W has an altered nucleotide-binding site but does not reveal conformational changes that would enable P_i_ egress, indicating that the R183 backdoor is not part of the dominant P_i_-escape path in wild-type actin. Although we cannot exclude the possibility that this release path is marginally sampled, we propose that, alternatively, P_i_ still mainly exits through the R177-N111 backdoor in the R183 mutants. In that scenario, the more negatively charged nucleotide-binding pocket in these mutants may explain why the negatively charged P_i_ is released slightly faster (Fig. 3b,c). Interestingly, it has previously been shown that p.R183W results in perturbed nucleotide release from actin monomers and impaired binding of non-muscle myosin-2A to the actin filament^41^. It is therefore expected that these altered molecular properties of actin-R183W are the major cause of defects in actin organization that lead to disease, rather than the slightly altered P_i_-release kinetics of this actin variant.

### Actin-N111S filaments display markedly shortened ADP-P_i_ caps

**Fig. 5:**
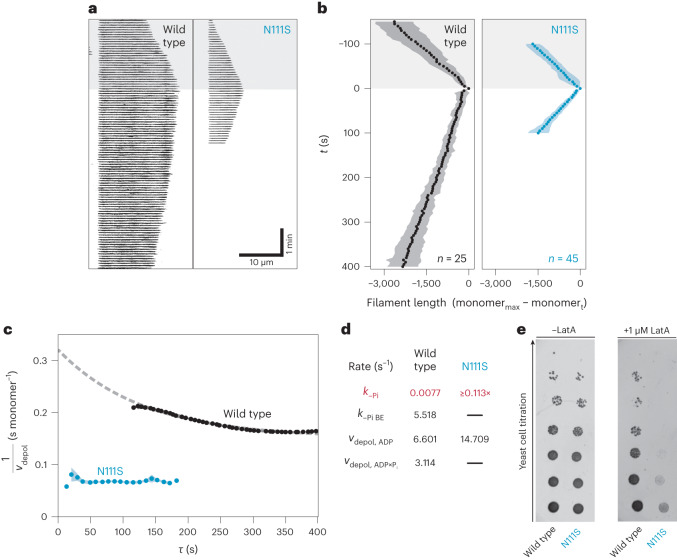
Effects of the N111S substitution on single actin filaments in vitro and on yeast growth in vivo. **a**, Timelapse TIRF imaging of single actin filaments (black, visualized by Lifeact-Alexa-Fluor-488) polymerized from spectrin-coated surfaces in microfluidic flow chambers. The vertical and horizontal axes indicate time and filament length, respectively. Polymerization and depolymerization phases are demarcated by the gray shaded and unshaded areas, respectively. **b**, Filament lengths tracked over the course of polymerization (gray shaded area) and depolymerization (unshaded area). Points indicate average filament length calculated from 25 and 45 filaments polymerized from either wild-type actin or actin-N111S, respectively. Shaded areas indicate the s.d. Wild-type and N111S filaments were analyzed from 6 and 12 independent experiments, respectively. *t*, time since depolymerization. Monomer_max_ and monomer_t_ are defined as the filament length in monomers at the maximum length and at time = *t*, respectively. **c**, The inverse of the instantaneous depolymerization velocity (1 / *v*_depol_), calculated from the average filament length in **b**, as a function of filament age ($\tau$) upon depolymerization. Points and the shaded area around them indicate average 1 / *v*_depol_ calculated from varying window sizes and the s.d., respectively. The dashed line indicates the fit to a kinetic model (see Methods). **d**, Rate constants of P_i_ release from the filament interior and barbed end (*k*_–Pi_ and *k*_–Pi BE_) and depolymerization velocities of ADP-P_i_ or ADP subunits from the filament end (*v*_depol,ADP_ and *v*_depol,ADP-Pi_) determined from fits obtained in **c**. The asterisk indicates the lower bound estimate for N111S (see Methods and Extended Data Fig. 5h). **e**, Growth phenotype assay of yeast expressing either the wild type or N111S variant of *S. cerevisiae* actin. The two yeast strains were grown in the absence or presence of 1 μM latrunculin A (LatA). Source data

Next, we hypothesized that actin-N111S, which releases P_i_ rapidly in bulk assays and adopts an open-backdoor conformation, should also display strong differences in nucleotide-state distribution at the single-filament level compared with wild-type actin. We reasoned that actin-N111S filaments should form drastically shortened ADP-P_i_ caps, which we tested in microfluidic flow-out assays using total internal reflection fluorescence (TIRF) microscopy (Fig. 5a and Supplementary Video 4). Filaments were elongated from surface-immobilized spectrin-actin seeds using either wild-type or actin-N111S and were then rapidly switched to depolymerization with buffer lacking soluble actin (see Methods). For wild-type β-actin, the speed of filament depolymerization after flow-out gradually increased over a few minutes before reaching the maximum rate (Fig. 5b,c). Since ADP-P_i_-bound actin depolymerizes at slower rates than does ADP-bound actin, this change is caused by the filament-depolymerizing region slowly maturing from an ADP-P_i_-rich to an ADP-rich composition through P_i_ release. The measured depolymerization velocities are well described by a kinetic model (Fig. 5d and Methods), with parameters similar to those previously measured in single-filament assays on skeletal α-actin^21^, as well as from our own bulk measurements (Figs. 3b and 5d). Strikingly, we observed that filaments grown from actin-N111S depolymerized at a high velocity (*v*_depol,ADP_ = 14.71 s^−1^) (Fig. 5b,c). More importantly, we found no appreciable change in the depolymerization velocity after buffer flow-out (Fig. 5c), indicating that the ADP-P_i_ to ADP-actin transition is very fast and was not captured within our experimental resolution. This allowed us to estimate a lower bound for the phosphate-release rate from the filament interior *k*_–Pi_ ≥ 0.113 s^−1^ for actin-N111S (Fig. 5d and Extended Data Fig. 5e). Hence, our data reveal that the rapid rate of P_i_ release indeed results in drastically shortened ADP-P_i_ caps in actin-N111S filaments.

To investigate the effect of the N111S substitution in vivo, we compared the growth rates of yeast strains expressing either wild-type or the N111S variant of *Saccharomyces* *cerevisiae* actin. Under normal conditions, we observed no major alterations in growth phenotype between the two strains (Fig. 5e). Accordingly, live-cell fluorescence imaging of the two yeast variants did not reveal large differences in the lifetime of endocytic actin patches (Extended Data Fig. 5i,j). However, when exposed to the toxin latrunculin A, which sequesters G-actin and accelerates F-actin depolymerization^44^, the yeast expressing actin-N111S displayed a dramatically reduced growth rate compared with that of yeast expressing wild-type actin (Fig. 5e). This high sensitivity to latrunculin-A-induced stress suggests that actin-N111S filaments are more labile and prone to depolymerization. Thus, the phenotype observed for *S. cerevisiae* actin-N111S in vivo is in line with our in vitro experiments investigating human β-actin-N111S, for which we observed faster P_i_-release and filament-depolymerization rates than were observed for wild-type actin.

Taken together, our experiments provide strong evidence that the first P_i_-egress pathway identified by enhanced-sampling MD, encompassing the R177-N111 backdoor, is the dominant P_i_-egress pathway from the F-actin interior. Hence, P_i_ is released from the core and barbed end of F-actin through similar exit routes, although core subunits require additional conformational rearrangements to transiently open the R177-N111 backdoor.

### The transient F-actin core state that allows for P_i_ release

**Extended Data Fig. 9:**
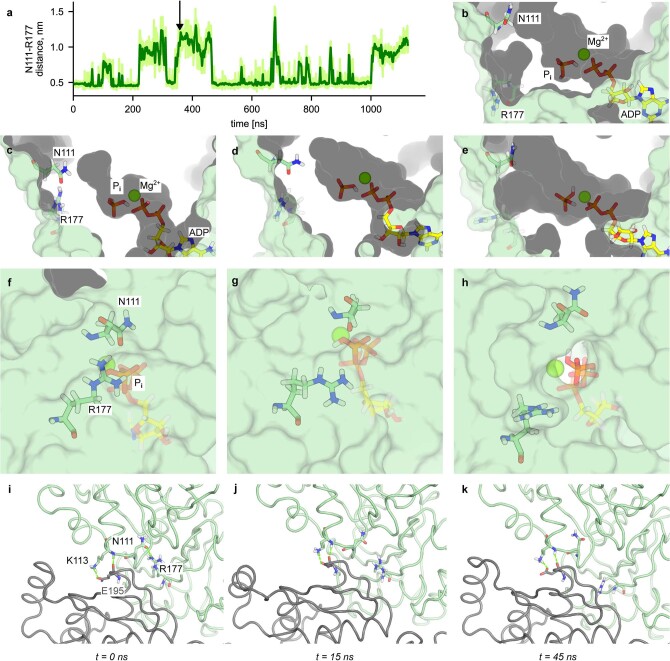
Structural dynamics of the R177-N111 backdoor in unbiased and steered MD simulations. **a,** Time-series of the R177CZ-N111CG distance (depicted in light green) in an unbiased MD simulation, revealing that the R177-N111 hydrogen bond reversibly breaks and re-forms in unbiased MD simulations. A distance larger than 0.6 nm indicates disruption of the hydrogen bond. For clarity, a centered 2 ns-moving average is shown in dark green. The arrow indicates the used time-frame to capture the F-actin conformation shown in panel b. **b**, Example configuration with a broken R177-N111 hydrogen bond sampled at 360 ns during the unbiased MD simulation. The surface representation indicates that the backdoor is closed despite the broken R177-N111 hydrogen bond. Solvent accessible area for P_i_ is depicted in dark grey. There is no accessible path for P_i_ to escape from the F-actin interior. **c-k** Snapshots from a representative SMD simulation (neutral meH73, replicate 1). Frames are the same as in Fig. 6. **c–e,** Solvent-accessible surface representation of the nucleotide binding site and R177-N111 backdoor, side view. The solvent-accessible volume is shown in dark grey. **f–h**, Solvent-accessible surface representation of the R177-N111 backdoor, external view. Note that in **h**, P_i_ is visible from the outside. **i–k,** Close-up on the inter-subunit contacts.

Finally, we sought to understand how P_i_ is released through the R177-N111 backdoor from the wild-type F-actin core. Our enhanced-sampling MD suggested that there are P_i_-escape pathways through the backdoor (Fig. 2c), but this protocol is aggressive by design, to explore diverse possible pathways, and may not reflect a realistic order of molecular motion. Therefore, we first ran unbiased MD simulations to investigate the backdoor conformation in F-actin. The simulations revealed the disruption and reformation of the R177-N111 interaction several times over a 1.1-μs period (Extended Data Fig. 9a). However, this never resulted in a functionally open backdoor (Extended Data Fig. 9b), indicating that more rearrangements are required for P_i_ to escape, and these were not captured in the timeframe of our unbiased simulations. This is in line with the very slow P_i_ release from wild-type actin (Fig. 3b).

**Fig. 6:**
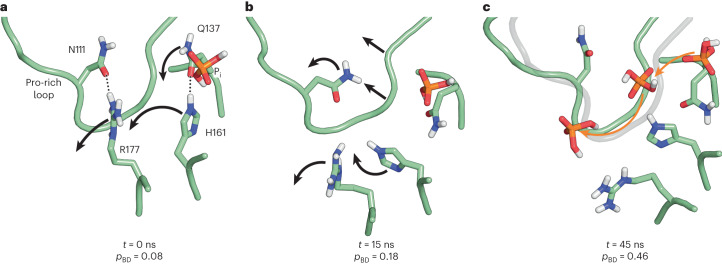
Backdoor opening mechanism revealed by SMD simulations. **a**–**c**, Selected frames from the SMD trajectory. Dotted lines indicate hydrogen bonds. Arrows indicate the side chain and backbone movements involved in the opening of the backdoor. Time *t* and P_i_-release propensity (*p*_BD_) along the trajectory are shown. In **c**, the orange arrows indicate the direction of P_i_ movement through the open R177-N111 backdoor observed in a representative enhanced sampling simulation.

**Extended Data Fig. 10:**
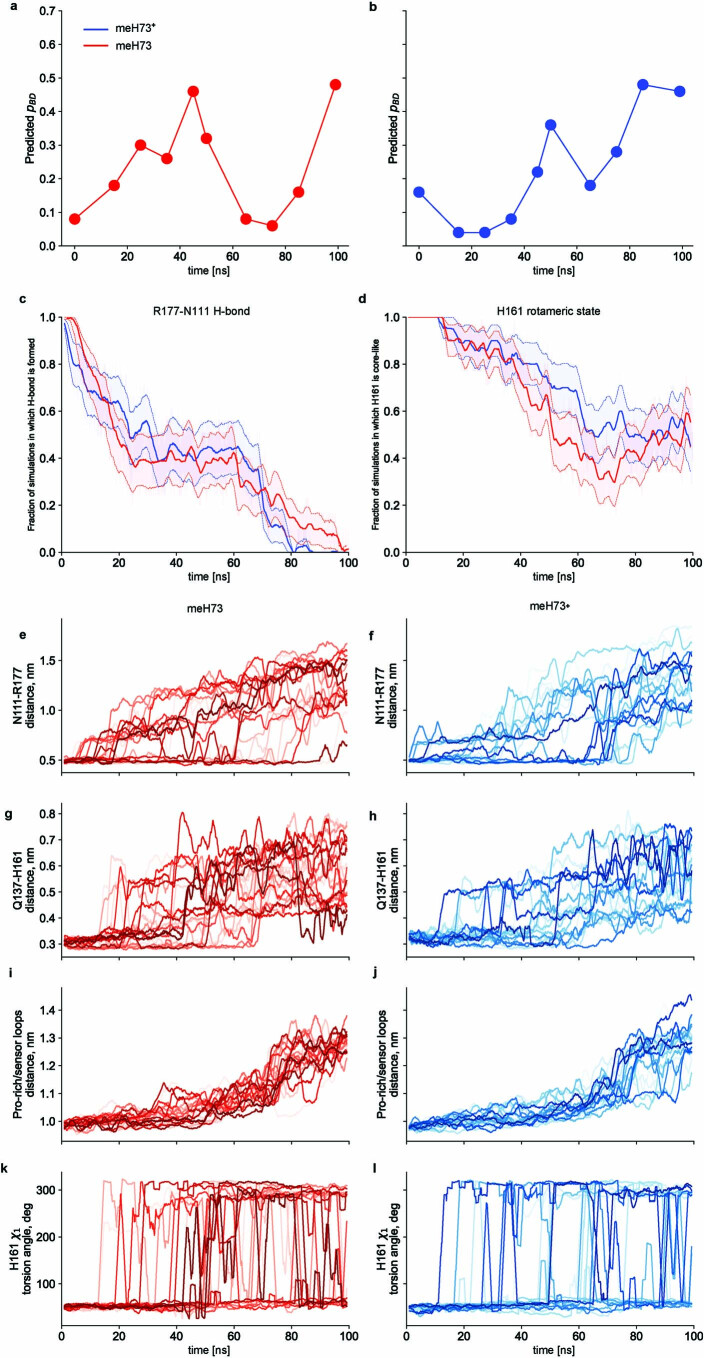
Evolution of key observables during SMD simulations. **a,** Predicted fraction _pBD_ of P_i_ egress through the R177-N111 backdoor along SMD replicate 1, with neutral meH73, as obtained by classifier analysis of the enhanced sampling trajectories. **b**, Predicted fraction _pBD_ of P_i_ egress through the R177-N111 backdoor along SMD replicate 1, with positively charged meHis73, as obtained by classifier analysis of the enhanced sampling trajectories. **c**, Survival analysis of the R177-N111 hydrogen bond in SMD simulations. For each protonation state of meH73, we computed the fraction of trajectories with the R177-N111 hydrogen bond still formed at time t. A distance cut-off of 0.6 nm between atoms CZ of R177 and CG of N111 was used to define a formed hydrogen bond. The solid lines represent the 2 ns-rolling average of the survival fraction. The dotted lines represent the 2 ns-rolling averages of the survival fraction ± SEM. The background overlays represent the raw values for the survival fraction ± SEM. **d**, Survival analysis of the rotameric state of H161. For each protonation state of meH73, we computed the fraction of trajectories with H161 in a core-like rotameric state. H161 side-chain rotameric states were defined by χ_1_ torsion angle being above or below 180 deg. Representation conventions are the same as for panel **c**. **e–l**. Evolution of structural observables during for all SMD simulation replicates. For clarity, 2 ns-moving averages are shown.

Therefore, to gain further mechanistic insights into the formation of this transient state of the F-actin core that allows for P_i_ release, we turned to steered-MD (SMD) simulations. We performed 40 SMD simulations of 100 ns each, in which selected regions of the backbone of an F-actin core protomer (with either charged or neutral methylated H73) were transitioned to the barbed-end conformation, which is a prototypical example of a functionally open backdoor (see Methods). Because this protocol steers the conformational transition in a quasi-deterministic fashion, structural rearrangements happen much faster than for stochastic backdoor opening. The majority of simulations showed movement of the Pro-rich loop and disruption of the R177-N111 hydrogen bond within 25 ns (Fig. 6a,b and Extended Data Figs. 9c–k and 10), representing the first major step of backdoor opening. The rotation of H161 to a G-actin-like conformation was also observed in many simulations and generally occurred after disruption of the R177-N111 interaction within 40–50 ns (Fig. 6b,c and Extended Data Fig. 10), while inter-subunit contacts within the filament remained intact (Extended Data Fig. 9k). We then evaluated the P_i_-release efficiency of structures extracted along these key timepoints in the SMD trajectories using our previously introduced metadynamics-based protocol. Although we did not observe a major effect of the charge of methylated H73 on backdoor opening, our simulations revealed a high probability (Fig. 6c, Extended Data Fig. 10a,b and Methods) that P_i_ would escape through the backdoor when the R177-N111 interaction was disrupted and the H161 side chain was flipped, suggesting that both events are required to stabilize the open-backdoor conformation. Thus, although only the backbone movement was steered in our SMD setup, the simulations elucidate the side chain rearrangements that lead to the transient opening of the backdoor in the F-actin core (Fig. 6).

## Discussion

**Fig. 7:**
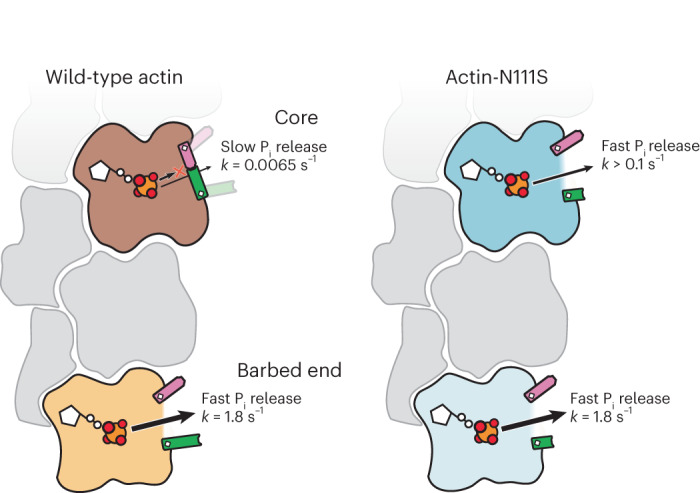
Cartoon model of P_i_ release from wild-type and N111S-F-actin. In wild-type F-actin, subunits that reside in the filament core predominantly adopt a closed-backdoor conformation and hence release P_i_ at slow rates. In the ultimate subunit at the barbed end, the backdoor is open, leading to 300-fold faster P_i_ release during actin depolymerization. In F-actin-N111S, the amino acid substitution results in an open backdoor in internal subunits, leading to increased P_i_-release rates.

By combining cryo-EM with in vitro reconstitution and MD simulations, we have elucidated how P_i_ is released from F-actin (Figs. 6 and 7) and that the hydrogen bonding network between the Pro-rich loop, sensor loop, and R177 strand forms the predominant backdoor. At the barbed end, the door for P_i_ release is open in the ultimate subunit, indicating that P_i_ can egress without large protein rearrangements. However, the backdoor is closed in F-actin core subunits and opens only transiently through a high-energy state. The opening of the door is likely initiated by a stochastic disruption of the hydrogen bonding network and is further induced by the change of rotameric position of H161 to a G-actin-like conformation. After P_i_ egress, H161 flips back to its original position and the hydrogen-bonding network is re-established, allowing F-actin to adopt its low-energy state with a closed backdoor. In actin-N111S, the backdoor is predominantly open in all subunits because the introduced S111 side chain is too short to maintain the interactions that keep the door closed. Hence, F-actin-N111S releases P_i_ rapidly upon polymerization, thereby drastically reducing the fraction of ADP-P_i_-bound subunits in the filament. Thus, our experimental and simulation data provide conclusive evidence that the P_i_-release rate from F-actin is controlled by steric hindrance through the backdoor.

Our barbed-end structure also provides further implications for actin polymerization. During the G- to F-actin transition, the side chain of residue H161 flips towards ATP, which triggers the relocation of water molecules near the nucleotide and, as a result, creates a favorable environment for ATP hydrolysis^7,8^. By contrast, at the barbed end, H161 adopts its G-actin-like rotameric position and points away from the nucleotide in the ultimate subunit, which suggests that, regardless of the growth velocity of the filament, the last subunit becomes ATP-hydrolysis-competent only when another actin subunit is added to the filament. This means that the full G- to F-form transition of an actin subunit should be considered a multi-step process, which not only encompasses the initial incorporation of that subunit into the filament, but also requires the subsequent binding of the next actin subunit. Formal proof for such a mechanism will require future investigations of the barbed end of F-actin in the ATP state. In general, we believe that investigating the structure of actin ends, either undecorated or bound by diverse classes of ABPs, will remain a prevalent theme of future research, guided by recent advances in actin-end structure determination by our lab and others^45–48^.

It has recently been shown that the presence of P_i_ at the nucleotide-binding site affects the bending structural landscape of F-actin^32^, which raises the question of whether backdoor opening could be affected by filament bending. Although backdoor opening involves a confined region of SD1 (the Pro-rich loop and sensor loop) and SD3 (R177-strand) in actin, the subdomains that display the largest displacements during filament bending are SD2 and SD4 (ref. ^32^). Accordingly, the backdoor is closed in all subunits of bent F-actin structures in the ADP (PDB 8D15) and ADP-P_i_ (PDB 8D16) states, suggesting that the backdoor state is not strongly affected by filament bending. In addition, if filament bending dramatically affected the P_i_-release kinetics of actin, one would expect P_i_ release to be cooperative, that is, actin subunits in the ADP state would bend more and thereby stimulate P_i_ release from neighboring subunits that adopt the ADP-P_i_ state. However, P_i_ release from the F-actin core is stochastic^21^. Therefore, we do not anticipate that filament bending majorly affects the dynamic opening and closing of the backdoor.

How are the kinetics of P_i_ release regulated in a complex cellular environment? Interestingly, the rates of P_i_ release from the filament interior observed in vitro appear to be slower than the turnover of some cellular actin structures, such as lamellipodial networks and endocytic patches^49^. This strongly suggests that P_i_ release in vivo is accelerated by ABPs through yet poorly defined mechanisms, which can be discussed in the context of our results. The P_i_-release backdoor opens towards the inner side of the filament, adjacent to where the two strands of the actin helix interact (Fig. 1d). This site is targeted by the small-molecule toxins phalloidin and jasplakinolide, which block backdoor opening and hence sterically inhibit P_i_ release^37–39^. Importantly, however, the backdoor and its immediate surroundings are not known to be directly engaged by many ABPs, presumably because molecules larger than phalloidin and jasplakinolide cannot easily enter the narrow cavity between the two actin strands. Accordingly, factors implicated in actin aging and turnover, such as ADF/cofilin and associated regulators like coronin^50,51^, all bind at the filament periphery^52–54^, suggesting that these ABPs can affect P_i_ release only allosterically. Such a mechanism has indeed been postulated for ADF/cofilin proteins, which efficiently bind and sever F-actin only following P_i_ release, but also accelerate the release of P_i_ from the filament^15,24^. Structurally, cofilin binding changes the tilt of F-actin^52,53^, resulting in rearrangements at the nucleotide-binding site that are incompatible with the presence of P_i_, rendering actin tilting and P_i_ binding mutually exclusive^8,53^. Hence, initial cofilin binding to the filament likely results in an actin conformation that releases P_i_ more rapidly. However, how such ABP-induced conformational changes promote backdoor opening, and how this relates to actin disassembly in cells, remains to be elucidated.

Finally, can our results explain how the N111S substitution leads to nemaline myopathy, a disease affecting α-actin in skeletal muscle? Although F-actin in striated muscle is expected to undergo less turnover than cytoplasmic actin isoforms, it is well established that actin-severing proteins, such as cofilins, are expressed in muscle sarcomeres to control actin (thin) filament length during sarcomerogenesis and actin turnover^55–57^. Since cofilins preferably sever ADP- over ADP-P_i_-bound F-actin, we propose that the ultrafast P_i_-release kinetics of actin-N111S may contribute to the pathophysiology in patients with this disease-linked mutation. Moreover, it will be interesting to study the effects of the N111S substitution in actin in non-muscle tissue. Because actin-N111S does not majorly populate the ADP-P_i_ state, this mutant could be a unique tool for investigating the role of the metastable ADP-P_i_ nucleotide state both in vivo and in vitro. We envision that our approach of studying mutants actin-R183W and actin-N111S, in which we combined biochemical experiments with high-resolution cryo-EM, will be instrumental in elucidating the molecular mechanisms of other disease-associated actin mutants. Specifically, visualizing the impact of substitutions on the atomic structure of F-actin will allow for the formulation of new hypotheses on how these affect cellular processes and how this is linked to disease. Therefore, this approach may ultimately contribute to the development of new therapeutic strategies for the treatment of human diseases that are characterized by mutations in actin genes.

## Methods

### DNA constructs and yeast strains

Throughout the manuscript, we used human β-actin amino acid numbering that is consistent with the numbering in the corresponding UniProt entry (P60709, ACTB_HUMAN), with the initiator methionine numbered as residue 1, even though this methionine is cleaved off during actin maturation. Hence, the amino acid numbers for β-actin used in our paper align with the sequences of mature human and rabbit skeletal α-actin, as well as *S. cerevisiae* actin, facilitating a direct comparison between all actin isoforms used in this study.

The plasmid for the expression of recombinant human β-actin (p2336 pFL_ACTB_C272A) was described previously^58^. All mutations were introduced *via* QuikChange PCR, using p2336 as template (Supplementary Table 1). All β-actin constructs contain the C272A substitution (including the protein referred to as wild type), as C272 is prone to oxidation in aqueous solutions^59^. The equivalent residue in human skeletal α-actin is also alanine. The FH2 domain of *Mus musculus* mDia1 (mDia1_FH2_) (amino acids 750–1163) was cloned into an pETMSumoH10 expression vector by Gibson assembly.

*S. cerevisiae* strains were grown in minimal medium containing yeast nitrogen base without amino acids (Difco) containing glucose and supplemented with tryptophane, adenine, histidine, and/or uracil if required. *S. cerevisiae* strains were transformed using the lithium-acetate method^60^. Yeast actin mutagenesis was performed as described previously^61^. Yeast viability in the presence or absence of latrunculin A was analyzed by drop test assays: fivefold serial dilutions of cell suspensions were prepared from overnight agar cultures by normalizing measurements of the optical density at 600 nm (OD_600_), then plated onto agar plates and incubated at 30 °C for 2 d. For live-cell imagining, yeast strains were transformed with plasmid 2477 (ref. ^62^) encoding Lifeact-mCherry under the constitutive promoter of *abp140*.

### Live-cell imaging of *S. cerevisiae* variants

Yeast cells expressing either wild-type actin or actin-N111S were cultured at 30 °C in minimal medium (2% glucose, 6.7 g L^–1^ yeast nitrogen base without amino acids, supplemented with 20 mg L^–1^ tryptophan and uracil) for at least one full cell cycle and were imaged when at an OD_600_ of between 0.5 and 1. Cells were seeded between the slide and coverslip, and all videos were acquired in less than 10 min after slide preparation. Cells were imaged using a spinning disk confocal device on the 3i Marianas system equipped with an Axio Observer Z1 microscope (Zeiss), a CSU-X1 confocal scanner unit (Yokogawa Electric Corporation), a Plan-Apochromat ×100/1.4 NA Oil Objective (Zeiss), and Orca Flash 4.0 sCMOS Camera (Hamamatsu), which was operated with Slidebook version 2022 software (3i). Temperature was maintained at 30 °C ± 1 °C at all times during imaging. Time-lapse images were collected every 500 ms for a total of 60 s of recording Lifeact intensity, which resulted in videos with 120 frames. To study in vivo actin dynamics, endocytic actin patches were detected and analyzed using the Fiji plugin TrackMate^63^. Patch detection was performed using a median filter and a Laplacian of Gaussian particle-detection algorithm for spots of 0.7 µm in diameter. Patches recognized by TrackMate were monitored, and the detected intensities that did not represent actin patches, that is, actin cables, were manually removed. Patch tracks were assigned using a simple LAP tracker. Resulting data were curated using a custom Python script to pre-process the data. Only patches in which the full lifespan was captured within the video were considered, and patches with a lifespan shorter than 4 s were not taken into account to avoid cable detection artifacts.

### Protein expression and purification

A mixture of native bovine cytoplasmic β-actin and γ-actin (from now on referred to as β,γ-actin) was purified from bovine thymus tissue, as described previously^26,46^. Bovine β and γ-actin both display 100% amino acid sequence identity to their corresponding human orthologs.

Human cytoplasmic β-actin variants were recombinantly expressed as fusion proteins, with thymosin β4 and a deca-His-tag fused to the actin C terminus^58,64^. β-actin was expressed in BTI-Tnao38 insect cells (CVCL_Z252, provided by S. Wohlgemuth and A. Musacchio, – Max Planck Institute of Molecular Physiology) using baculovirus infection. At ~48 h after infection, the cells were pelleted (5,000*g*, 10 min) and stored at −80 °C until use. On the day of purification, the insect cells were thawed, resuspended in lysis buffer containing 10 mM Tris pH 8, 50 mM KCl, 5 mM CaCl_2_, 1 mM ATP, 0.5 mM TCEP, and cOmplete protease inhibitor (Roche), and were subsequently lysed using a fluidizer. The lysed cells were subjected to ultracentrifugation (100,000*g*, 25 min) to remove cell debris. The supernatant was filtered and then loaded onto a 5 ml HisTrap FF crude column (Cytiva). The column was washed with 10 column volumes of lysis buffer supplemented with 20 mM imidazole. The fusion protein was eluted from the column using a linear imidazole gradient (20 mM to 160 mM in 10 min). The eluted fusion protein was then dialyzed overnight in G-buffer (5 mM Tris pH 8, 0.2 mM CaCl_2_, 0.1 mM NaN_3_, 0.5 mM ATP, 0.5 mM TCEP), followed by incubation with chymotrypsin (1/250 wt/wt ratio of chymotrypsin to actin) for 20 min at 25 °C to cleave thymosin β4 and the deca-His-tag from the β-actin. Proteolysis was terminated by the addition of 0.2 mM PMSF (final concentration), and the mixture was applied to a clean HisTrap FF crude column. The flowthrough was collected and β-actin was polymerized overnight by the addition of 2 mM MgCl_2_ and 100 mM KCl (final concentrations). The next morning, actin filaments were pelleted by ultracentrifugation (210,000*g*, 2 h) and resuspended in G-buffer. β-actin was depolymerized by dialysis in G-buffer for 2–3 d. Afterwards, the protein was ultracentrifuged (210,000*g*, 2h) to remove any remaining filaments. The supernatant was, if necessary, concentrated in a 10-kDa concentrator (Amicon) to 2–3 mg ml^–1^, flash frozen in liquid nitrogen in 50-µl aliquots, and stored at −80 °C until further use.

Native rabbit α-actin was purified from muscle acetone powder and labeled with pyrene (N-1-pyrene-Iodoacetamide, Sigma). Ten grams of rabbit muscle acetone powder was mixed with 200 ml G-Buffer and stirred for 30 min at 4 °C. Later, the solution was centrifuged at 16,000*g* in a GSA rotor for 1 h at 4 °C to separate the supernatant containing actin from the debris. Actin polymerization was initiated in the supernatant by adding polymerization buffer (50 mM KCl, 1.5 mM MgCl_2_, 1 mM EGTA, 10 mM imidazole pH 7) and an additional 0.1 mM ATP. The solution was allowed to polymerize at room temperature for 1 h while being stirred, after which the solution was moved to 4 °C to polymerize for another hour. Later, the concentration of KCl in the solution was increased to 800 mM, and it was stirred for another 30 min. After a total of 2.5 h of polymerization, the solution was centrifuged at 96,000*g* in a Ti45 rotor for 1 h at 4 °C and the actin pellet was resuspended in F-Buffer (2 mM Tris pH 8.0, 50 mM KCl, 1.5 mM MgCl_2_, 1 mM EGTA, 10 mM imidazole, 0.1 mM CaCl_2_, 0.2 mM ATP, 0.01% NaN_3_, 0.5 mM TCEP). Polymerized actin was reduced by the addition of 1 mM TCEP, and the mixture was centrifuged at 266,000*g* in a TLA-110 rotor for 30 min at 4 °C. The resulting actin pellet was resuspended in labeling buffer (50 mM KCl, 1.5 mM MgCl_2_, 1 mM EGTA, 10 mM imidazole pH 7, 0.2 mM ATP), to which pyrene was added in five times molar excess of actin, and incubated on ice for 2 h. The reaction was quenched by the addition of 10 mM DTT and was dialyzed in G-buffer to depolymerize actin.

For the purification of heterodimeric DNase I–actin complexes, deoxyribonucleaseI from bovine pancreas (DNase I, Serva, cat. no. 18535.02) was dissolved at a concentration of 666 μM (20 mg ml^–1^) in 1×KMEI Buffer (0.5 mM ATP, 1 mM TCEP, 50 mM KCl, 1.5 mM MgCl_2_, 1 mM EGTA, 10 mM imidazole pH 7) containing 1× complete protease inhibitors and 1 mM PMSF. Then, 4 ml of 90 µM filamentous β,γ-actin was mixed with 1.1 ml of 666 μM DNase I, resulting a 1:2 molar ratio, and depolymerized by dialysis for 1 week against G-buffer. The dialyzed sample was centrifuged for 30 min at 80,000 r.p.m. in a TLA-110 rotor, and the supernatant was gel filtered into G-Buffer over a Superdex 200 16/600 column (Cytiva). Fractions corresponding to the heterodimeric DNase I–actin complex were pooled, concentrated, and stored at 4 °C for up to 3 months.

mDia1_FH2_ was expressed with an amino-terminal 10×His-SUMO3-tag in *Escherichia coli* BL21 Star pRARE cells for 16 h at 18 °C. The cells were lysed in Lysis Buffer-v2 (50 mM NaH_2_PO_4_ pH 8.0, 400 mM NaCl, 0.75 mM β-mercaptoethanol, 15 μg ml^–1^ benzamidine, 1× complete protease inhibitors, 1 mM PMSF, DNase I) and the protein was purified by IMAC using a 5-ml HisTrap column. The protein was eluted using Elution Buffer (50 mM NaH_2_PO_4_ pH 7.5, 400 mM NaCl, 400 mM imidazole, 0.5 mM β-mercaptoethanol) in a gradient and the 10×His-SUMO3-tag was directly cleaved using SenP2 protease overnight. After cleavage, proteins were desalted into lysis buffer and recirculated over a 5-ml HisTrap column, followed by gel filtration over a Superdex 200 16/600 into Storage Buffer (20 mM HEPES pH 7.5, 200 mM NaCl, 0.5 mM TCEP, 20% glycerol), concentrated, flash frozen in liquid nitrogen, and stored at −80 °C.

Spectrin-actin seeds were purified as described previously^65,66^. The purified spectrin-actin seeds were biotinylated by addition of a fivefold molar excess of maleimide-PEG_2_-biotin. After incubation for 30 min on ice, biotinylated spectrin-actin seeds were separated from free, unreacted maleimide-PEG_2_-biotin by two rounds of desalting over NAP-5 columns. Glycerol was added to 50% (vol/vol), and biotinylated spectrin-actin seeds were stored at −20 °C until use.

### Synchronous measurement of actin polymerization and P_i_ release in bulk assays

On the day of the assay, aliquots of all purified β-actin variants (frozen as G-actin) were thawed and centrifuged at 100,000*g* for 20–30 min to remove aggregates. To ensure that all variants were in exactly the same buffer, we exchanged the buffer to G-buffer-v2 (5 mM Tris pH 8, 0.2 mM CaCl_2_, 0.1 mM NaN_3_, 0.1 mM ATP, 0.5 mM TCEP) using Micro Bio-Spin Chromatography Columns (Bio-Rad). For each measurement, 40 μl G-actin solution containing 30.5 μM unlabeled β-actin variant, 0.5 μM pyrene-labeled, wild-type α-actin, and 15 μM P_i_-sensor (MDCC-labeled phosphate binding protein, Thermo Fisher Scientific) was prepared. We confirmed that the presence of trace amounts (1.5%) of pyrene-labeled, wild-type α-actin, which releases P_i_ with slow kinetics, did not substantially contribute to the overall readout by the phosphate sensor, which was dominated by P_i_ release from the actin mutant present in vast excess (98.5%) (Extended Data Fig. 5a). Thirty-six microliters of G-actin solution was then mixed with 4 μl 10×ME (5 mM EGTA pH 7.5, 1 mM MgCl_2_) and incubated at room temperature for 2 min, in order to exchange the ATP-associated divalent cation from Ca^2+^ to Mg^2+^. We then took 36 μl of this solution and mixed it with 64 μl polymerization buffer (16 mM HEPES pH 7, 160 mM KCl, 3 mM MgCl_2_, 1.5 mM EGTA, 38 μM P_i_-sensor, 160 nM spectrin-actin seeds, 0.1 mg ml^–1^ β-casein, 0.1 mM ATP, 0.5 mM TCEP) in a quartz cuvette to start the experiment. This yielded final concentrations of 10 μM actin (1.5% pyrene-labeled), 100 nM spectrin-actin seeds, 100 mM KCl, and 30 μM P_i_-sensor. The spectrin-actin seeds were added to ensure rapid polymerization in order to (1) minimize potential differences in the polymerization kinetics between the β-actin variants (Extended Data Fig. 5b) and (2) create a pronounced separation between the time courses of polymerization and P_i_ release, at least in the case of wild-type actin. Measurements were taken in a spectrofluorometer (PTI QM-6) under constant excitation at 365 nm and synchronous monitoring at 1-s intervals of pyrene (wavelength *λ* = 410 nm) and MDCC (wavelength *λ* = 455 nm) fluorescence intensities, which report on actin polymerization and phosphate release, respectively.

### Determination of P_i_-release rates from bulk assays

Timecourses of pyrene or MDCC fluorescence from individual experiments were first normalized by subtracting the minimal signal at the beginning of the experiment and dividing by the maximal signal at saturation at *t* = 900 s. Observed rates of actin polymerization (*k*_poly_) were then determined by fitting time courses of normalized pyrene fluorescence from individual experiments by a mono-exponential function in Origin Pro version 9.0G:

$$I\left(t\right)=1-{e}^{-{k}_{\mathrm{poly}}t}$$

with *I*(*t*) being the normalized fluorescence intensity as a function of time. Average *k*_poly_ values for each actin variant were calculated from three independent experiments (Extended Data Fig. 5b). To determine *k*_–Pi_, we first averaged time courses of normalized MDCC fluorescence from three individual experiments for each actin variant. The averaged data were fitted by a simple kinetic model using the KinTeK Explorer software (version 6.3):

$${\mathrm{A}}\mathop{\longrightarrow}\limits^{k_{\mathrm{poly}}} {\mathrm{B}} \mathop{\longrightarrow}\limits^{k_{{\hbox{-}}\mathrm{P}_{\mathrm i}}}_{{\longleftarrow}\atop k_{+\mathrm{P}_{\mathrm i}}}{\mathrm{C}}+{\mathrm{P}}_{\mathrm{i}}$$

with A being monomeric ATP-actin, B being filamentous ADP-P_i_-actin, and C being filamentous ADP-actin. The model contains three kinetic parameters, two of which were fixed. The first-order rate of polymerization (*k*_poly_), which formally is the sum of the rates of both polymerization and ATP hydrolysis, was fixed to the experimentally measured polymerization rate for each actin variant (see above). The second-order association rate constant for binding of inorganic phosphate (*k*_+Pi_) was fixed to 0.000002 µM^−1^ s^−1^, as measured previously for wild-type α-actin and assumed to be the same for all actin variants^11,27^. This assumption likely does not hold, because the chosen substitutions can be anticipated to similarly accelerate both release and binding of P_i_. However, we determined that *k*_+Pi_ can be varied by more than 1,000-fold without majorly affecting the obtained first-order rate of phosphate release (*k*_–Pi_). More importantly, we can exclude the possibility that rebinding of P_i_ contributes substantially under our experimental conditions: P_i_ was (1) generated only in minor amounts (10 µM) during the course of the assay and (2) potently sequestered by the phosphate sensor that was present in molar excess (30 µM) and that binds P_i_ with 10,000-fold-higher affinity (dissociation constant (*K*_D_ ) = 0.1 µM) (ref. ^67^) than that of actin (*K*_D_ = 1.5 mM) (refs. ^11,27^).

For the N111S mutant, we could not determine the exact rate constant of P_i_ release in this manner, because the average observed rate of P_i_ release slightly exceeded the average observed polymerization rate (Fig. 3b). This should formally not be possible because the latter has to precede the former, and the reason for this inversion remains unknown. To nonetheless obtain a conservative estimate for the increase in the P_i_-release rate in this case, we carried out kinetic simulations in KinTek Explorer to systematically explore the dependence of the observed P_i_-release reaction kinetics on the rate enhancement of P_i_ release (Extended Data Fig. 5c,d). This showed that a rate enhancement of P_i_ release by more than 15-fold is required for the observed P_i_ release rate to fall within the error margin of the observed polymerization rate. Hence, we consider 0.1 s^−1^ the lower bound for the rate of P_i_ release for the N111S mutant.

### Preparation of functionalized glass slides

Functionalized glass slides coated with 5% biotin-PEG and 95% hydroxy-PEG were prepared as has been described^68^. Briefly, high-precision glass coverslips (22 × 60 mm, 1.5H) were asymmetrically cut at one corner using a diamond pen to distinguish the functionalized surface from the non-functionalized one. Glass slides were cleaned by incubation in 3 M NaOH solution for 15 min, rinsed in water, and incubated in freshly prepared Piranha solution (3:2 mixture of 95–97% sulfuric acid and 30% hydrogen peroxide) for 30 min. Slides were rinsed with water to remove residual acid and were then air dried using nitrogen gas. Dried glass slides were sandwiched with 3 drops of GOPTS (3-glycidyloxypropyl trimethoxysilane) and then stored in closed Petri dishes, which were further incubated in an oven at 75 °C for 30 min. Sandwiched glass slides were rinsed in acetone and separated with a pair of tweezers. Following separation, the glass slides were rinsed again in fresh acetone. Separated glass slides were air dried with nitrogen gas and placed in pre-warmed Petri dishes with their functionalized surface facing up. The functionalized surfaces were sandwiched with 75 µL of a 150 mg ml^–1^ mixture of 95% hydroxy-amino-PEG (ɑ-hydroxy-ω-amino PEG-3000) and 5% biotinyl-amino-PEG (ɑ-biotinyl-ω-amino PEG-3000) dissolved in acetone. Sandwiched glass surfaces were incubated in closed Petri dishes in an oven at 75 °C for 4 h. Following incubation, the glass slides were separated with a pair of tweezers, rinsed multiple times in water, and air dried using nitrogen gas.

### Preparation of microfluidic devices

Microfluidic devices were prepared by casting polydimethylsiloxane (PMDS) onto a silicon mold designed to incorporate up to four inlets and one outlet. A mixture of PDMS and its curing agent (10:1 mass ratio, SYLGARD 184 Silicone Elastomer Kit) was thoroughly mixed and poured onto the silicon mold. The PDMS cast was degassed in a desiccator under vacuum for 3 h to remove bubbles from the cast. After removal of bubbles, the cast was incubated in an oven at 75 °C for another 4 h to complete the curing process. On completion of the curing process, the PDMS microfluidic devices were cut from the cast, rinsed with isopropanol, and air dried with nitrogen gas.

The functionalized glass cover slips and the flow surface of the microfluidic devices were plasma cleaned at 0.35 mbar pressure and 80% ambient air for 3 min. Parts of the functionalized glass cover slips that would eventually line up with the observation chamber on the microfluidic device were protected from plasma treatment by placing blocks of PDMS on the region. The microfluidic device was sealed by placing the PDMS block onto the functionalized glass surface and incubating at 75 °C for 1 h.

### Microfluidic experiments and TIRF microscopy of single filaments

The surface of a flow channel was prepared by first passivating the surface to avoid nonspecific interactions, then coating the biotinylated surface with streptavidin, and finally coating the surface with biotinylated spectrin-actin seeds. Channels were passivated by washing with 1× KMEI buffer supplemented with 1% Pluronic F-127, 0.1 mg ml^–1^ β-casein, and 0.1 mg ml^–1^ κ-casein. Passivated surfaces were coated with streptavidin by flowing in 1× KMEI containing 0.75 nM streptavidin. Streptavidin-coated surfaces were then coated with spectrin-actin seeds by washing with 1× KMEI containing 10 nM biotinylated spectrin-actin seeds. Each of the above steps was interleaved by a wash step with in 1× KMEI after each step. All steps were carried out at flowrates of 20 µl min^–1^ for 5 min each, except the streptavidin flow step which was 2 min long. After the surfaces were coated with spectrin seeds, polymerization was started by flowing in assay buffer (0.1 mg ml^–1^ β-casein, 1 mM ATP, 1 mM DABCO, 20 mM β-mercaptoethanol, 100 mM KCl, 1.5 mM MgCl_2_, 1 mM EGTA, 20 mM HEPES) containing ~2.5 µM profilin:actin (2.71 µM wild-type β-actin or 2.50 µM β-actin-N111S) and 50 nM Alexa-Fluor-488-Lifeact for visualization of filament growth. The profilin:actin amount was varied to equalize minor differences in the polymerization velocity between the two actin variants. Image acquisition was started immediately after the profilin:actin buffer flow was initiated. Depolymerization was initiated after about 3–5 min of polymerization by stopping the flow of profilin:actin and starting the wash with depolymerization buffer (0.1 mg ml^–1^ β-casein, 1 mM ATP, 1 mM DABCO, 20 mM β-mercaptoethanol, 100 mM KCl, 1.5 mM MgCl_2_, 1 mM EGTA, 20 mM HEPES, 50 nM Alexa-Fluor-488-Lifeact).

Image acquisition was carried out using a TIRF microscope with a ×60 objective and a ×1.5 zoom lens under TIRF conditions. Time-lapse images were acquired every 5 s with 2% laser power, 2-s exposure time, and an electronic gain setting of 150, and emitted light was filtered through an emission filter of 525 nm with a 50-nm bandpass. All images were acquired at a bit-depth of 16 bits.

### Filament tracking and data analysis

Timelapse TIRF microscopy images of actin filaments were first denoised using the non-local means algorithm implemented with a custom Python script. Lengths of single actin filaments were tracked in time with ImageJ using the JFilament plugin^69^ with the following parameters: alpha = 15, beta = 10, gamma = 20,000, weight = 0.5, stretch = 2,000, deform iterations = 200, spacing = 1.0, smoothing = 1.01, curve type = open. Foreground and background values were assigned by selecting regions of the filament and the surrounding background, respectively.

Some of the filaments stopped depolymerizing at different time points during the depolymerization phase; therefore, we restricted our analysis to time scales under which the filament depolymerization was uninterrupted (Extended Data Fig. 5g). Pauses in the depolymerization of single filaments have been reported to be a result of photo-induced dimerization and were extensively characterized in a previous study^70^. Our tracked filaments demonstrate pausing behavior similar to those observed previously, wherein the cumulative distribution of the pause probability fits a sigmoidal model as a function of time^70^ (Extended Data Fig. 5g). Filament lengths tracked from different filaments were averaged and used to calculate depolymerization velocity. Instantaneous depolymerization velocities were calculated by performing local linear regression of varying sizes centered around a time point. We found that the combined standard prediction error for the three fit parameters of the kinetic model displays a broad minimum as a function of window size (Extended Data Fig. 5e). Altering the window size within this minimum (sizes 17–29) yields correspondingly smaller or larger delay times in the velocity data, with marginal effects on the obtained fit parameters (Extended Data Fig. 5f). We systematically chose window sizes that reliably predict fit parameters with an average prediction standard deviation error of less than 10% (Extended Data Fig. 5e,f). Instantaneous velocities calculated for each time point were then averaged over different window sizes. An exponential decay function of the form *y* = *ae*^−*bx*^ + *c* was used to fit 1 / *v*_depol_ versus filament age ($\tau$) curves, where:

$$y=\frac{1}{{v}_{{\rm{depol}}}}$$

$$b={k}_{{\hbox{-}}\text{Pi}}$$

$$c=\frac{1}{{v}_{{\rm{depol}},{\rm{ADP}}}}$$

$$a+c=\frac{1}{{v}_{{\rm{depol}},{\rm{ADP}}\times {\rm{Pi}}}}$$

Filament age during the depolymerization phase was calculated as follows:

$$\tau =t+\frac{{L}_{0}\,-\,{L}_{t}}{{v}_{{pol}}}$$

Where *t* is the time since depolymerization, *L*_0_ is the filament length at the start of depolymerization, *L*_t_ is the filament length at time *t*, and *v*_pol_ is the polymerization velocity.

The barbed-end P_i_-release rate was calculated as follows:

$${{\kappa }}_{-\text{Pi BE}}={v}_{{\rm{depol}},{\rm{ADP}}}\left(\frac{{{\kappa }}_{\text{of}\,\text{f}}^{\text{ADP}-\text{Pi}}-{v}_{{\rm{depol}},{\rm{ADP}}\times{\rm{Pi}}}}{{v}_{{\rm{depol}},{\rm{ADP}}\times {\rm{Pi}}}-{v}_{{\rm{depol}},{\rm{ADP}}}}\right)$$

Where *k*_−Pi_ and *k*_−Pi BE_ are the phosphate-release rates from the interior of the filament and the barbed end, respectively. ${v}_{\text{depol}}$, ${v}_{{\rm{depol}},{\rm{ADP}}}$, and ${v}_{{\rm{depol}},{\rm{ADP}}\times {\rm{Pi}}}$ are the observed depolymerization rate, depolymerization rate of ADP-actin subunits, and depolymerization rate of ADP-P_i_-actin subunits, respectively. ADP-P_i_-actin subunits can dissociate from the filament in two different ways; either as actin–ADP-P_i_ with a rate of ${{\kappa}}_{\text{off}}^{{\text{ADP}}-{\text{Pi}}}$ or first by releasing the P_i_ at a rate of *k*_–Pi BE_ and then dissociating at a rate of ${v}_{{\rm{depol}},{\rm{ADP}}}$ (ref. ^21^). For our calculations, we fixed ${{\kappa }}_{\text{off}}^{\text{ADP}-\text{Pi}}$ to 0.2 s^−1^, as measured previously^27^.

We estimated lower bounds for the P_i_-release rate of actin-N111S by assuming that ${v}_{\text{ADP}-\text{Pi}-\text{depol}}$ was either equal to or twice that of wild-type actin. The latter assumption was motivated by the observation that the ${v}_{{\rm{depol}},{\rm{ADP}}}$ rate of the N111S mutant was about 2.2 times that of wild-type actin (Fig. 3d and Extended Data Fig. 5h).

### Preparation of short filaments for cryo-EM

The in vitro polymerization of actin filaments from purified G-actin generally results in long filaments (>500 nm). Cryo-EM imaging of such filaments at high magnification typically yields 0–2 actin ends per micrograph, which does not allow for the averaging of enough end particles to obtain a high-resolution structure. Hence, previous studies have relied on capping protein (CP) as a potent actin nucleator and barbed-end capper to obtain short filaments^45,46^. However, CP binds the F-actin barbed end with high affinity, and we aimed to reconstruct an undecorated barbed end. We therefore designed a new protocol in which we used two other ABPs to create short filaments: DNase I and mDia1_FH2_, which act as pointed-end capper and actin nucleator, respectively. Eighty micromolar β/γ-actin–DNase I complex was mixed with 2 μM free β/γ-actin and 25 μM mDia1_FH2_ (final concentrations) in a total volume of 50 μl G-buffer. The sample was then incubated with 5.6 μl 10× ME on ice for 1 min to exchange the ATP-associated divalent cation in actin from Ca^2+^ to Mg^2+^. Actin polymerization was induced by the addition of 6.2 μl 10× KMEH (100 mM HEPES pH 7.0, 1000 mM KCl, 20 mM MgCl_2_, 10 mM EGTA), resulting in a salt concentration of 100 mM KCl and 2 mM MgCl_2._ After the mixture was incubated in a room-temperature water bath for 60 s, we added 1 μl phalloidin (in DMSO) to a final concentration of 80 μM. The sample was placed on ice for another 30 min, and was then injected onto a Superdex 200 increase 5/150 column (Cytiva) pre-equilibrated in 10 mM imidazole pH 7.1, 100 mM KCl, 2 mM MgCl_2_ and 1 mM EGTA on an ÄKTA Micro system (Cytiva). The eluted filament fractions were collected and directly used for cryo-EM grid preparation within 30 min.

### Cryo-EM sample preparation of actin-R183W and actin-N111S

The recombinant human β-actin filaments with the N111S or R183W substitution were prepared similarly to what has described previously for native rabbit skeletal α-actin filaments^8^. Frozen G-actin aliquots (53 μM of actin-N111S, 58 μM of actin-R183W) in G-buffer were thawed and centrifuged at 100,000*g* for 20–30 min to remove aggregates. Fifty microliters of G-actin was then mixed with 5.6 μl 10× ME and incubated for 5 min on ice to exchange the ATP-associated divalent cation from Ca^2+^ to Mg^2+^. Actin polymerization was induced by moving the sample to room temperature, followed by the addition of 6.2 μl 10× KMEH, resulting in a final salt concentration of 100 mM KCl and 2 mM MgCl_2._ The two mutants were polymerized for 1 h at room temperature, followed by 1–2 h on ice. The long incubation period following polymerization ensured that both β-actin variants adopted the ‘aged’ ADP nucleotide state. The formed filaments were subsequently centrifuged at 200,000*g* for 30 min, and the F-actin pellet was resuspended in 1× KMEH (10 mM HEPES pH 7.1, 100 mM KCl, 2 mM MgCl_2_, 1 mM EGTA) supplemented with 0.02% Tween 20 (vol/vol), to a final concentration of ~15 μM F-actin. The resuspended material was used for cryo-EM grid preparation within 1 h.

### Cryo-EM grid preparation

All cryo-EM grids were prepared using the same protocol; 2.8 µl of F-actin sample was applied to a glow-discharged R2/1 Cu 300 mesh holey-carbon grid (Quantifoil). Excess solution was blotted away, and the grids were plunge frozen in liquid ethane or a mixture of liquid ethane and propane using a Vitrobot Mark IV (Thermo Fisher Scientific, operated at 13 °C). The short β/γ-actin filaments, which allowed for the structural determination of the barbed end, were blotted with a blotting force of 0 for 3 s. The long filaments of recombinant β-actin with the N111S or R183W substitution were blotted with a blotting force of −20 for 8 s.

### Cryo-EM grid screening and data collection

The barbed-end dataset was collected on a 200 kV Talos Arctica cryo-microscope (Thermo Fisher Scientific) with a Falcon III direct electron detector (Thermo Fisher Scientific) operated in linear mode. Using EPU (Thermo Fisher Scientific), 1,316 movies were collected at a pixel size of 1.21 Å in 40 frames, with a total electron exposure of ~56 e^−^ Å^–2^. The defocus values in EPU were set from −2.7 to −1.2 μm.

The β-actin-N111S (9,516 movies) and β-actin-R183W (7,916 movies) datasets were collected on a 300 kV Titan Krios G3 microscope (Thermo Fisher Scientific) equipped with a K3-direct electron detector (Gatan) and a post-column BioQuantum energy filter (Gatan, slit width 15 eV). For both datasets, movies were obtained in super-resolution mode in EPU at a pixel size of 0.3475 Å in 60 frames (total electron exposure of ~70 e^−^ Å^–2^). The defocus values in EPU were set from −2.0 to −0.7 μm.

### Cryo-EM image processing

All datasets were pre-processed on the fly using TranSPHIRE^71^. Within TranSPHIRE, gain and beam-induced motion correction was performed using UCSF MotionCor2 v1.3.0 (ref. ^72^). The super-resolution mode collected data (β-actin-N111S and β-actin-R183W datasets) were binned twice during motion correction (resulting pixel size, 0.695 Å). CTFFIND4.13 (ref. ^73^) was used to estimate the contrast transfer function, and particles or filament segments were picked using SPHIRE-crYOLO^74^.

For the β/γ-actin barbed-end dataset, particles were picked using SPHIRE-crYOLO in regular single-particle mode^74^, resulting in 252,982 particles. The picked particles were binned four times and extracted in a 96 × 96 box (pixel size, 4.84 Å) in RELION 3.1.0 (ref. ^75^). The extracted particles were then imported into CryoSPARC v.3.3.2 (ref. ^76^), which was used for the majority of image processing of this dataset. Importantly, the particles were processed as regular single particles without any applied helical symmetry or restraints. We first performed a 2D classification into 100 classes, thereby removing 11,275 junk particles. The remaining 241,709 particles were subjected to two rounds of heterogeneous refinements. In the first round, we supplied a reference of the pointed end and a reference of a complete filament core. Through this approach, we removed all particles that represented the pointed end (106,675 particles) and kept all particles that were classified into the full filament. For the second round, the remaining particles (135,034 particles) were again heterogeneously refined against two references: a full filament map and a barbed-end map. This allowed for the removal of 72,877 filament core particles and the isolation of 62,157 barbed-end particles. These barbed-end particles were then un-binned (pixel size, 1.21 Å) and subjected to non-uniform refinement, resulting in a map at a resolution of 4.07 Å. We then converted the particles to RELION (using csparc2star.py) for Bayesian polishing^77^. Afterwards, we re-imported them into CryoSPARC and ran iterative 2D classifications and three-dimensional (3D) classifications without image alignment to remove any remaining junk particles. Following one extra round of Bayesian polishing, the final 43,618 barbed-end particles were non-uniformly refined, with the per-particle defocus estimation option switched on, to a reconstruction of 3.51-Å resolution. To further improve the density of subunits at the barbed end, we created a soft mask around the first four actin subunits (from the end) and ran a local refinement in CryoSPARC. The resulting cryo-EM density map was refined to a slightly lower resolution (3.59 Å) but showed an improved local resolution for the penultimate and ultimate subunits at the barbed end.

For the β-actin-N111S and β-actin-R183W datasets, filament segments were picked using the filament mode of SPHIRE-crYOLO^78^, with a box distance of 40 pixels between segments (corresponding to 27.8 Å) and a minimum of six boxes per filament. This yielded 2,001,281 and 1,569,882 filament segments (from now on referred to as particles) for the N111S and R183W datasets, respectively, which were extracted (384 × 384 box) and further processed in helical SPHIREv1.4 (ref. ^79^). The processing strategy was essentially the same as that reported in our previous work^8^. Briefly, the extracted particles were first 2D classified using ISAC2 (refs. ^80,81^) in helical mode, and all non-protein picks were discarded. The particles were then refined using meridien alpha, which imposes helical restraints to limit particle shifts to the helical rise (set to 27.5 Å) to prevent particle duplication, but does not apply helical symmetry^71^. For both datasets, the first refinement was performed without a mask using EMD-15109 as the initial reference, low-pass filtered to 25 Å. From the reconstruction that we obtained, a soft mask was created that covered 85% of the filament length within the box (326 pixels in the *z* direction). Masked meridien alpha refinements then yielded density maps at resolutions of 2.9 Å (N111S dataset) and 3.0 Å (R183W dataset). Subsequently, the particles of both datasets were converted to be readable by RELION through sp_sphire2relion.py. In RELION, the particles were subjected to Bayesian polishing (two times) and CTF refinements^82^, followed by a 3D classification without image alignment into eight classes. We selected the particles that classified into high-resolution classes and removed duplicates, which yielded a final set of 1,756,928 and 1,386,604 particles for the N111S and R183W datasets, respectively. Finally, these particles were refined from local searches (sampling 0.9°) with solvent flattening Fourier shell correlations (FSCs) in RELION, yielding reconstructions at resolutions of 2.30 Å (N111S) and 2.28 Å (R183W). RELION was also used to estimate the local resolutions of each density map.

### Model building, refinement and analysis

The barbed-end β/γ-actin structure was modeled as β-actin. Of the four amino acid substitutions between β- and γ-actin, three represent N-terminal amino acids (D2, D3 and D4 in β-actin; E2, E3 and E4 in γ-actin) that are not visible in the density map owing to flexibility. Accordingly, the only other residue (V10 in β-actin, I10 in γ-actin) that is different between both isoforms was modeled as valine. To construct an initial model for human β-actin, chain C of the 2.24-Å cryo-EM structure of rabbit skeletal α-actin in the Mg^2+^-ADP state (PDB 8A2T—94% sequence identity to human β-actin, all water molecules removed) was rigid-body fitted in the third actin subunit from the barbed end (A_2_) in the cryo-EM density map using UCSF ChimeraX-1.5 (ref. ^83^). Rabbit skeletal α-actin residues were substituted with the corresponding residues of human β-actin in Coot^84^. The resulting model was then also fitted in the densities for the A_0_, A_1_, and A_3_ actin subunits. The model for the cyclic toxin phalloidin was taken from PDB 6T1Y (ref. ^39^) and rigid-body fitted into the density map. The barbed structure was iteratively refined by manual model building in Coot and Phenix real-space refine^85^ with applied Ramachandran and rotamer restraints. As described in the manuscript, the model of the ultimate A_0_ actin subunit had to be substantially altered from the starting models.

The model of recombinant β-actin with the N111S substitution was obtained through a similar approach; chain C of PDB 8A2T (including all water molecules) was fitted into the central actin subunit of the density map. After substitution of all α-actin specific amino acids to the corresponding β-actin residues, introduction of the N111S substitution, and further manual model building in Coot, the resulting model was fitted in four more actin subunits (chains A, B, D, E) in the density map. The filament was modeled as a pentamer to capture the full interaction interface of the central subunit with its four neighboring subunits. All water molecules were first manually built, inspected and adjusted in the central subunit, and were then copied to the other chains with non-crystallographic symmetry (NCS). Because the local resolution was worse at the periphery of the reconstruction, we removed water molecules that displayed poor corresponding cryo-EM density in the non-central actin chains. The model was refined in Phenix real-space refine with NCS restraints but without Ramachandran and rotamer restraints. The model of β-actin-R183W was constructed and refined in the same way, except that the β-actin-N111S structure was used as the starting model for model building. A summary of the refinement quality of the structures is provided in Table 1. Figures depicting cryo-EM density maps and protein structures were prepared using ChimeraX-1.5.

### Preparation of structural models for MD simulations

All-atom models of the core and barbed end of F-actin were prepared from, respectively, PDB 8A2S (F-actin core in the Mg^2+^-ADP-P_i_ state, ref. ^8^) and PDB 8OI6 (the F-actin barbed-end structure presented in this study). Missing residues 46–48 in the D-loop were built de novo with MODELLER v10.2 (ref. ^86^). To ensure that simulations could be compared between structural states, the barbed-end structure was back-mutated from human β-actin to human/rabbit α-actin using MODELLER. The missing C-terminal residues (363–375) of the ultimate subunit in the barbed-end structure were modeled using the corresponding resolved residues in the adjacent subunit as template. Phalloidin molecules were removed in the barbed-end model. Hydrogen atoms were added with CHARMM c43b2 (ref. ^87^). The P_i_ ion was modeled in the H_2_PO_4_^−^ state, using previously established parameters^88^. This is consistent with a recent quantum-mechanical analysis of ATP hydrolysis by F-actin^7^, and also promotes sampling of P_i_-egress events by reducing the strength of electrostatic interactions^36^. Protonation states of histidine residues were determined with ProPka^89^. Histidine 73 was modeled as a protonated methyl-histidine (meH73^+^). For the F-actin core, a separate model with a neutral methyl-histidine 73 (meH73) was also prepared. Non-histidine titratable residues were modeled in their standard state. For the actin core models, structural water molecules were kept. As the barbed-end structure was determined in the ADP-bound state, we added the P_i_ ion in the same position relative to ADP as seen in the actin core structure. Structural models were embedded in an orthorhombic simulation box (20 nm × 10.4 nm × 8.8 nm for actin core, 18.8 nm × 11.6 nm × 8.8 nm for barbed end), solvated with TIP3P water molecules, and supplemented with Na^+^ and Cl^−^ ions to ensure electroneutrality and reach a total salt concentration of 150 mM. CHARMM, VMD^90^, and scripts obtained from CHARMM-GUI^91^ were used for model preparation. Each model was then energy-minimized for 5,000 steps of steepest descent, with harmonic position restraints (force constant 4,184 kJ mol^–1^ nm^–^²) applied on backbone atoms.

### MD simulation parameters

All simulations were performed with GROMACS 2021.5 (refs. ^92,93^) patched with the February 2022 version of the colvars module^94^. Energetics were described with the CHARMM36m force-field^95^. Van der Waals forces were smoothly switched to zero between 1.0 nm and 1.2 nm. Long-range electrostatics was treated by the particle mesh ewald (PME) method. The cut-off between short-range and long-range electrostatics was initially set to 1.2 nm, and then was adjusted automatically by GROMACS to improve simulation performance. The length of covalent bonds involving hydrogen atoms was constrained with LINCS^96^. The leapfrog integrator, with a 2-fs timestep, was used for molecular dynamics time integration. An average temperature of 310 K was maintained with the v-rescale thermostat^97^ (Berendsen for equilibration^98^); an average pressure of 1 bar was maintained with the Parrinello–Rahman barostat^99^ (Berendsen for NPT equilibration, that is, at a fixed number of particles N, average pressure P and average temperature T). Minimized systems were equilibrated under active backbone restraints by 1 ns of NVT simulation (that is, at a fixed number of particles N, box volume V and average temperature T) followed by 1 ns of NPT simulation. For production simulations, backbone restraints were turned off, and the filament was kept parallel to the box using a harmonic restraint on the orientation quaternion (force constant 10^5^ kJ mol^–1^), as implemented in colvars. MD trajectories were visualized and analyzed with VMD, Pymol (Schrödinger) and MDAnalysis^100^.

### Metadynamics protocol to sample P_i_ release

To efficiently sample structurally diverse P_i_-release events from actin, we developed an enhanced sampling protocol based on simulating ‘swarms’ of short metadynamics trajectories^36,101^. Metadynamics applies a history-dependent potential on a user-defined collective variable to make previously visited configurations more unstable, thus enhancing the sampling. Here, we apply metadynamics on P_i_ to drive it out of the active site. For this purpose, we concurrently applied separate conventional metadynamics biases on the three Cartesian coordinates *x*_P_, *y*_P_, and *z*_P_ of the phosphorus atom of the P_i_ ion, expressed in the internal reference frame of the actin filament. The bias was applied only on the P_i_ ion of the relevant actin subunits, namely the ultimate subunit in the barbed end and the central subunit in the F-actin core. The bias deposition frequency was set to 0.125 ps^−^^1^, the bias strength to *W* = 1 kJ mol^–1^, and the Gaussian potential s.d. to 0.05 nm. These parameters ensure the capture of P_i_-escape events. We stress that they are not suited for the evaluation of the converged free-energy profile along a P_i_-egress path, which was not the purpose of our simulations. Each swarm was composed of 50 independent 10-ns-long metadynamics simulations launched from the same structure. Swarms were analyzed by visual inspection and machine learning.

### Clustering and analysis of P_i_-egress paths

To get a broad picture of the main P_i_-egress paths sampled by metadynamics, we used hierarchical clustering in path space. For this purpose, P_i_ was considered to have escaped when the distance of the P atom from its initial position in the frame of the actin filament exceeded a cut-off value of 1.4 nm, and frames following escape were discarded for the rest of the analysis. We clustered escape paths as follows. First, each P_i_-egress trajectory (represented by the time-series of the *x*_P_, *y*_P_, *z*_P_ coordinates defined above) was smoothed using B-spline interpolation. Then, 100 equally spaced points were extracted along the fitted splines for each trajectory, resulting in 50 discretized paths in Cartesian space per swarm. Finally, the Euclidean distance matrix between the 50 discretized paths was computed. From this matrix, we performed hierarchical clustering of P_i_-egress paths. To visualize the paths corresponding to each cluster, we first computed each cluster’s centroid path as the cluster average in discretized path space. Then, we used the closest cluster member to the centroid as this cluster’s representative path. We found that a user-defined value of four clusters was sufficient to uncover the main sterically accessible P_i_-egress paths from actin. Spline interpolation was performed with SciPy^102^, and clustering was performed with scikit-learn^103^. To complement the agnostic clustering analysis, we also evaluated the fraction of paths within a swarm for which P_i_ egress took place through the R177-N111 backdoor. We visually inspected the individual P_i_ -egress trajectories and labeled the trajectories in which escape through the R177-N111 backdoor was observed. Escape through the R177-N111 backdoor was defined as P_i_ passing in between the side chains of R177 and N111, or in their close vicinity.

### Unbiased molecular dynamics of actin core

To obtain insights into the conformational dynamics of actin, a 1.1-µs unbiased MD simulation of the F-actin core (meH73^+^) was performed. For the central actin subunit, a soft positional restraint (force constant 625 kJ mol nm^–2^) was applied on the coordinates *x*_P_, *y*_P_, and *z*_P_ of the P_i_ ion of the central subunit to keep it close to the post-hydrolysis position. We analyzed the opening state of the R177-N111 backdoor by measuring the distance between R177CZ and N111CG atoms (N111-R177 distance) along the simulation trajectory.

### Classifier model to detect P_i_ egress through the R177-N111 backdoor

We trained a logistic regression binary classifier to automatically detect trajectories in which P_i_ egress happens through the R177-N111 backdoor. Our classifier takes as input a featurized P_i_-egress trajectory from a metadynamics simulation, and returns 1 if this trajectory is identified as escaping through the R177-N111 backdoor, and returns 0 otherwise. To make the model insensitive to differences in absolute Cartesian coordinates between simulations of different structures, we developed an input trajectory featurization based on the distances of P_i_ to key residues during egress. First, to deal with escape trajectories of unequal time lengths, we computed for each trajectory the longitudinal path collective variable *s* (pathCV^104^) ranging from 0 (P_i_ in the post-hydrolysis position) to 1 (P_i_ escapes). Thus, *s* provides a common rescaled time that enables the comparison of trajectories of different lengths. The pathCV calculation was performed with an in-house Python script. The *λ* parameter was set to 325 nm^−2^, and the reference path was obtained by B-spline interpolation of the *x*_P_, *y*_P_, and *z*_P_ time-series, and then was discretized into 30 equally spaced points. For each simulation frame of a given P_i_-egress trajectory, we then computed *s*, along with distances between the P atom of P_i_ and the CA atom of 48 residues involved in typical egress pathways detected in the clustering and/or visual analyses (residues 12–18, 71–74, 106–116, 120, 134–138, 141, 154–161, 177, 183, 300–301, 334–338, 370, 374). Distances were turned into Boolean contact values using a distance cut-off of 0.75 nm. Then, contact values along egress paths were grouped into four bins according to the pathCV *s* value, and the average P_i_ contact occupancies per bin and per residue were computed. This procedure yielded a P_i_ contact map along discretized rescaled time, which has the same 48 × 4 size for every P_i_-egress trajectory regardless of the actual time to P_i_ escape. Each contact map was finally flattened into a column vector whose entries were used as input features for the classifier. Training of the classifier was performed using a manually labeled data set of 300 P_i_-egress trajectories, including 70 positives. This data set was randomly split into training and test sets in a 75%/25% proportion using class balancing to preserve the proportion of true positives. The classifier was trained on the training set, and the confusion matrix was evaluated on the test set. scikit-learn was used for these tasks. Out of 75 labeled trajectories in the test set, the classifier predicted 52 true negatives, 6 false positives, 16 true positives and 1 false negative.

### Steered MD simulations

To simulate the opening of the R177-N111 backdoor from the filament interior, we used SMD to drive the central actin core subunit towards the barbed-end configuration and away from the actin core configuration. For this purpose, we applied a harmonic restraint (force constant 334,124 kJ mol^–1^ nm^–2^ or 800 kcal mol^–1^ Å^–2^) moving with constant velocity on the Δr.m.s. deviation (Δr.m.s.d.), that is, the difference in r.m.s.d. between actin core and barbed-end structures. The biasing potential $U$ acting on atomic configuration $x$ at time $t$ was:

$$\begin{array}{l}U(x,t)=\frac{1}{2}k\left({{\mathrm{r.m.s.d}}}(x,\text{actin core})-{{\mathrm{r.m.s.d}}}(x,\text{barbed end})\right.\\\left.\qquad\qquad-{{\mathrm{r.m.s.d}}}(\text{actin core},\text{barbed end})-{vt}\right)^{2}\end{array}$$

with *v* = 0.0022 nm ns^–1^ and where r.m.s.d.(*x*, actin core) refers to the r.m.s.d. of atomic configuration *x* with respect to the actin core structure, r.m.s.d.(*x,* barbed end) refers to the r.m.s.d. of atomic configuration *x* with respect to the barbed-end structure and r.m.s.d. (actin core, barbed end) refers to the r.m.s.d. of the actin core structure with respect to the barbed end structure. R.m.s.d. values were computed on the Cα atoms of residues 8–21, 29–32, 35–38, 53–68, 71–76, 79–93, 103–126, 131–145, 150–166, 169–178, 182–196, 203–216, 223–232, 238–242, 246–250, 252–262, 274–284, 287–295, 297–300, 308–320, 326–332, 338–348, 350–355, 359–365 and 367–373. To mimic the influence of a long actin filament in cellular conditions, static r.m.s.d. restraints (force constant 4,184 kJ mol^–1^ nm^–2^) were applied separately on the Cα atoms of two pairs of lateral actin subunits flanking the central subunit. To decouple backdoor opening from P_i_ movement, a positional harmonic restraint was applied on the P atom Cartesian coordinates *x*_P_, *y*_P_, and *z*_P_ (force constant 625 kJ mol^–1^ nm^–2^). Forty SMD simulations were run (20 with meH73^+^ and 20 with meH73). The following structural observables were computed along each trajectory: N111-R177 distance (see above), sensor/Pro-rich loops distance (defined as the distance between the centers of geometry of Cα atoms of sensor-loop residues 70–75, and Cα atoms of Pro-rich loop residues 105–115), H161-Q137 distance (defined as the distance between H161-atom NE2 and Q137-atom OE1) and the H161 side chain torsion angle (${\chi }_{1}$). To evaluate the capacity of structures sampled by SMD to release P_i_ through the R177-N111 backdoor, frames were extracted at *t* = 0 ns (equilibrated structure), *t* = 15 ns, 25 ns, 35 ns, 45 ns, 50 ns, 65 ns, 75 ns, 85 ns, and 100 ns. Then, each of these frames was used to launch a swarm of 50 metadynamics simulations to probe P_i_ release according to the protocol described above. A static Δr.m.s.d. restraint with the same force constant and reference value as the SMD potential was applied to preserve the configuration of the actin core subunit. For each swarm, all trajectories were analyzed with the logistic regression classifier. Finally, the proportion *p*_BD_ of trajectories for which backdoor escape through R177-N111 was predicted was computed for each swarm.

### Reporting summary

Further information on research design is available in the Nature Portfolio Reporting Summary linked to this article.

## Online content

Any methods, additional references, Nature Portfolio reporting summaries, source data, extended data, supplementary information, acknowledgements, peer review information; details of author contributions and competing interests; and statements of data and code availability are available at 10.1038/s41594-023-01101-9.

### Supplementary information

Supplementary Information Supplementary Table 1

Reporting Summary

Peer Review File

Supplementary Video 1 Structure of the F-actin barbed end reveals an open backdoor for P_i_ release in the ultimate subunit. This video shows the cryo-EM density and atomic model of the barbed end of the actin filament. Zooms are shown for the P_i_-release backdoor conformation in actin subunits A_2_ and A_0_, followed by a morph between the two conformations.

Supplementary Video 2 Metadynamics-based simulations reveal the P_i_-release path from the actin filament interior. This video depicts an enhanced sampling simulation of P_i_ escape from the F-actin interior through the R177-N111 backdoor. For clarity, only selected amino acid residues of actin are shown.

Supplementary Video 3 Structural comparison between wild-type- and N111S-actin filaments. This video shows a comparison between wild-type actin (PDB 8A2T) and N111S-actin (PDB 8OID) filaments in the ADP nucleotide state. Zooms are shown for the nucleotide binding site and the P_i_-release backdoor conformation in both structures.

Supplementary Video 4 Timelapse TIRF imaging of wild-type-actin (top) and actin-N111S (bottom) filaments during polymerization and depolymerization. This video depicts wild-type (top) and actin-N111S (bottom) filaments during polymerization in the presence of ~2.5 µM profilin-actin, and subsequent depolymerization. Filaments were visualized with 50 nM Alexa-Fluor-488-labeled Lifeact. The timeseries has been aligned to the start of the depolymerization phase in both experiments.

### Source data

Source Data Figs. 3 and 5 and Extended Data Fig. 5 Tab Fig. 3b: All individual data points for the experimental and simulated data in the graphs depicted in Fig. 3b. Tab Fig. 5b: All data points for the graph depicted in Fig. 5b. Tab Fig. 5c: All experimental and fitted data points for the graph depicted in Fig. 5c. Tab Extended Data Fig. 5a: All data points for the graph depicted in Extended Data Fig. 5a. Tab Extended Data Fig. 5b: All values for the data points shown in the graph in Extended Data Fig. 5b. Tab Extended Data Fig. 5c: All individual data points for the experimental and simulated data in the graph depicted in Extended Data Fig. 5c. Tab Extended Data Fig. 5d: All values for the data points shown in the graph in Extended Data Fig. 5d. Tab Extended Data Fig. 5e: All values for the data points shown in the graph in Extended Data Fig. 5e. Tab Extended Data Fig. 5f: All individual data points for the experimental and simulated data in the graph depicted in Extended Data Fig. 5f. Tab Extended Data Fig. 5g: All experimental and fitted data points for the graph depicted in Extended Data Fig. 5g. Tab Extended Data Fig. 5h: All experimental and fitted data points for the graph depicted in Extended Data Fig. 5h. Tab Extended Data Fig. 5j: All values for the data points shown in the graph in Extended Data Fig. 5j.

## Data Availability

The cryo-EM maps generated in this study have been deposited in the Electron Microscopy Data Bank (EMDB) under accession codes (dataset in brackets): EMD-16887 (β/γ-actin barbed end), EMD-16888 (F-actin-R183W) and EMD-16889 (F-actin-N111S). These depositions include sharpened and unsharpened maps, unfiltered half-maps and the masks used for refinements. The associated protein models have been deposited in the Protein Data Bank (PDB) with accession codes 8OI6 (β/γ-actin barbed end), 8OI8 (F-actin-R183W) and 8OID (F-actin-N111S). The following previously published protein models were used for data analysis and comparisons: 8A2S, 8A2T and 2V52. We used EMD-15109 as a 3D model for the first refinements of F-actin-R183W and F-actin-N111S. The sequence of human β-actin (P60709, ACTB_HUMAN) was retrieved from UniProt. MD simulation models and protocols, MD simulation datasets, and Jupyter notebooks to reproduce the analyses reported in Extended Data Figs. 4, 9 and 10 have been deposited in Zenodo (10.5281/zenodo.7765025). All other materials are available from the corresponding authors upon request. Source data are provided with this paper.

## Code availability

All scripts used for data analysis of single-filament assays can be retrieved from https://github.com/iamankitroy/Actin-Pi-Release.
