# Supplementary material for: Molecular mechanisms of inorganic-phosphate release from the core and barbed end of actin filaments
Source: Nat Struct Mol Biol. 2023 Sep 25;30(11):1774–85. doi: 10.1038/s41594-023-01101-9 (PMC10643162; doi:10.1038/s41594-023-01101-9)
Supplement: Supplementary file 1 — Supplementary Table 1 [file 41594_2023_1101_MOESM1_ESM.pdf]

nature structural & molecular biology

Article

https://doi.org/10.1038/s41594-023-01101-9

Molecular mechanisms of inorganic-phosphate release from the core and barbed end of actin filaments

In the format provided by the authors and unedited

Supplementary Table 1. Used constructs and primers in this study.

Construct name	Template Vector	Used Primers* (cloning strategy)
2920 pFL_ACTB(beta actin cytosolic)_linker_T4b_10His C272A R183W	2336 pFL_ACTB(beta actin cytosolic)_linker_T4b_10His C272A (ref.[58])	CCTGGCTGGCTGGGACCTGACTG (FW), TCCAGACGCAGGATGGCATGG (RV) (QuikChange PCR)
2921 pFL_ACTB(beta actin cytosolic)_linker_T4b_10His C272A R183G	2336 pFL_ACTB(beta actin cytosolic)_linker_T4b_10His C272A (ref.[58])	CCTGGCTGGCGGGGACCTGACTG (FW), TCCAGACGCAGGATGGCATG (RV) (QuikChange PCR)
2925 pFL_ACTB(beta actin cytosolic)_linker_T4b_10His C272A N111S	2336 pFL_ACTB(beta actin cytosolic)_linker_T4b_10His C272A (ref.[58])	GGCCCCCCTGTCCCCCAAGGCCAAC (FW), TCGGTCAGCAGCACGGGG (RV) (QuikChange PCR)
pB969 pRS313 Actin N111S (*S. cerevisiae)*	p1387 pRS313 Actin (ref.[61])	AAGCTCCAATGAGCCCTAAATCAAA (FW), CAGGAAACAGCTATGACC (RV), TTCGTGATAAGTGATAGTG (FW) TTTGATTTAGGGCTCATTGGAGCTT (RV) (Two-step overlap PCR)
825 PETMSumoH10_mDia1(FH2)	pDCMV-mNeongreen-mDia1FH1-2 (ref.[26])	GACGTGTTCCAGCAACAGACCGGTGGATT AACCCCCAAAAAAGTTTATAAGC (FW) ctcagtggtggtggtggtgGTGCTCGAGTTATTACTG CTTCTCCAGTCGCTCTTTTTC (RV) (Gibson Assembly)

*FW and RV stand for forward and reverse, respectively.
