# Supplementary material for: Molecular mechanisms of inorganic-phosphate release from the core and barbed end of actin filaments
Source: Nat Struct Mol Biol. 2023 Sep 25;30(11):1774–85. doi: 10.1038/s41594-023-01101-9 (PMC10643162; doi:10.1038/s41594-023-01101-9)
Supplement: Supplementary file 2 — Reporting Summary [file 41594_2023_1101_MOESM2_ESM.pdf]

nature portfolio

Corresponding author(s): Gerhard Hummer, Peter Bieling, Stefan Raunser

Last updated by author(s): Aug 2, 2023

Reporting Summary

Nature Portfolio wishes to improve the reproducibility of the work that we publish. This form provides structure for consistency and transparency in reporting. For further information on Nature Portfolio policies, see our Editorial Policies and the Editorial Policy Checklist.

Statistics

For all statistical analyses, confirm that the following items are present in the figure legend, table legend, main text, or Methods section.

n/a	Confirmed	
☐	☒	The exact sample size (*n*) for each experimental group/condition, given as a discrete number and unit of measurement
☐	☒	A statement on whether measurements were taken from distinct samples or whether the same sample was measured repeatedly
☒	☐	The statistical test(s) used AND whether they are one- or two-sided<br>*Only common tests should be described solely by name; describe more complex techniques in the Methods section.*
☒	☐	A description of all covariates tested
☐	☒	A description of any assumptions or corrections, such as tests of normality and adjustment for multiple comparisons
☐	☒	A full description of the statistical parameters including central tendency (e.g. means) or other basic estimates (e.g. regression coefficient) AND variation (e.g. standard deviation) or associated estimates of uncertainty (e.g. confidence intervals)
☒	☐	For null hypothesis testing, the test statistic (e.g. *F*, *t*, *r*) with confidence intervals, effect sizes, degrees of freedom and *P* value noted<br>*Give P values as exact values whenever suitable.*
☒	☐	For Bayesian analysis, information on the choice of priors and Markov chain Monte Carlo settings
☒	☐	For hierarchical and complex designs, identification of the appropriate level for tests and full reporting of outcomes
☒	☐	Estimates of effect sizes (e.g. Cohen's *d*, Pearson's *r*), indicating how they were calculated

Our web collection on statistics for biologists contains articles on many of the points above.

Software and code

Policy information about availability of computer code

Data collection	Cryo-EM data was collected using the commercially available software EPU version 2.8 (Thermofisher Scientific).<br><br>Molecular dynamics simulations were performed using GROMACS 2021.5 and colvars 2022-02-20-dev.
Data analysis	Cryo-EM data collection was monitored and preprocessed on the fly using TranSPHIRE version 1.5.13. The preprocessing steps in TranSPHIRE involved gain and drift correction using UCSF MotionCor2 v1.3.0, CTF estimation with CTFFIND4 v4.1.13, and particle picking using SPHIRE-crYOLO v1.5.8. All cryo-EM data were further processed using helical SPHIRE v1.4, RELION v3.1.0 and CryoSPARC v3.3.2. Protein model building was performed in COOT v0.9.8.1 and the models were refined using phenix real-space refine v1.20.1-4487-000. Protein models were validated within the phenix suite v1.20.1-4487-000. Figures and videos that depict cryo-EM density maps and protein structures were prepared using UCSF ChimeraX v1.5.<br><br>Bulk biochemical data was analyzed using Origin Pro version 9.0G. Kinetic simulations were carried out in KinTek Explorer version 6.3.<br><br>All scripts used for data analysis of single-filament assays can be retrieved from https://github.com/iamankitroy/Actin-Pi-Release/. Single filament microfluidic timelapse images were denoised using custom python script, NL-Means_Denoise.py. Single filaments were tracked with the JFilament 1.02 plugin on Image 2.9.0/1.53t. Calculation of filament length and alignment of length and depolymerization time was performed with analyze_filament_tracking.py. Instantaneous depolymerization velocity and their fits were computed with filament_instant-depolVelocity_analysis.py. Kinetic constants were calculated for WT filaments using calc_kinetic-constants.py and estimated for N111S mutant filaments using mutant-kinetic-contants-estimation.ipynb. All scripts were executed with Python 3.9.16 with the following version of modules: Scikit-Image 0.19.3, Numpy 1.24.2, SciPy 1.10.1 and Pandas 1.5.3. All single molecule data were plotted in R 4.2.2 with ggplot 3.3.6.<br><br>Molecular dynamics simulations were analyzed using Python 3.9.12, MDAnalysis 2.3.0, numpy 1.20.3 and pandas 1.5.2. Machine learning was performed using scikit-learn 1.1.3, scipy 1.9.3. Figures that visualize molecular dynamics trajectories were created using Python 3.9.12 and VMD 1.9.4. Model rebuilding for MD was performed using MODELLER v10.2. Hydrogen atoms were added with CHARMM c43b2.

Live cell imaging movies were analyzed using Python 3.10.10, pandas 2.0.0 and numpy 1.23.5. Figures were created using GraphPad Prism 9.

For manuscripts utilizing custom algorithms or software that are central to the research but not yet described in published literature, software must be made available to editors and reviewers. We strongly encourage code deposition in a community repository (e.g. GitHub). See the Nature Portfolio guidelines for submitting code & software for further information.

Data

Policy information about availability of data

All manuscripts must include a data availability statement. This statement should provide the following information, where applicable:

- Accession codes, unique identifiers, or web links for publicly available datasets
- A description of any restrictions on data availability
- For clinical datasets or third party data, please ensure that the statement adheres to our policy

The cryo-EM maps generated in this study have been deposited in the Electron Microscopy Data Bank (EMDB) under accession codes (dataset in brackets): EMD-16887 (β/γ-actin barbed end), EMD-16888 (R183W-F-actin) and EMD-16889 (N111S-F-actin). These depositions include sharpened and unsharpened maps, unfiltered half-maps and the masks used for refinements. The associated protein models have been deposited in the Protein Data Bank (PDB) with accession codes 8OI6 (β/γ-actin barbed end), 8OI8 (R183W-F-actin) and 8OID (N111S-F-actin). The following previously published protein models were used for data analysis and comparisons: 8A2S [https://doi.org/10.2210/pdb8A2S/pdb], 8A2T [https://doi.org/10.2210/pdb8A2T/pdb] and 2V52 [https://doi.org/10.2210/pdb2V52/pdb]. We used EMD-15109 [https://www.ebi.ac.uk/emdb/EMD-15109] as 3D model for the first refinements of R183W- and N111S-F-actin. The sequence of human β-actin (P60709, ACTB_HUMAN) was retrieved from UniProt [https://www.uniprot.org/uniprotkb/P60709/entry]. MD simulation models and protocols, MD simulation datasets and Jupyter notebooks to reproduce the analyses reported in Extended Data Figs. 4, 9, 10 have been deposited in Zenodo (10.5281/zenodo.7765025). All other materials are available from the corresponding authors upon request.

Field-specific reporting

Please select the one below that is the best fit for your research. If you are not sure, read the appropriate sections before making your selection.

☒ Life sciences ☐ Behavioural & social sciences ☐ Ecological, evolutionary & environmental sciences

For a reference copy of the document with all sections, see nature.com/documents/nr-reporting-summary-flat.pdf

Life sciences study design

All studies must disclose on these points even when the disclosure is negative.

Sample size

Sample sizes for the three cryo-EM datasets presented in this study: For the barbed end dataset, 1,316 micrographs were collected. 262,982 total particles were picked and 43,618 particles were used for the final reconstruction. For the R183W-F-actin dataset, 7,916 micrographs were collected. 1,569,882 total particles were picked and 1,286,604 particles were used for the final reconstruction. For the N111S-F-actin dataset, 9,516 micrographs were collected. 2,001,281 total particles were picked and 1,756,928 particles were used for the final reconstruction.
These sample sizes of ~1000-10,000 micrographs are common in the cryo-EM field for obtaining high-resolution protein structures, see for example Oosterheert et al. Nature (2022): https://doi.org/10.1038/s41586-022-05241-8.

The in vitro bulk phosphate release assays were performed as three independent experiments per actin variant. The sample size of n=3 is common for in vitro assays with purified proteins, see for example Belyy et al. Plos Biol. (2020): https://doi.org/10.1371/journal.pbio.3000925

For single filament biochemical assays, we similarly adhered to established practices in the field, by analyzing more than 20 filaments per experimental condition, see Jegou et al Plos Biol (2011): https://doi.org/10.1371/journal.pbio.1001161.g002. Such number is sufficient, because hundreds of depolymerizing events occur per filament depolymerization phase.

The yeast drop test assays were performed in three independent experiments. Live cell imaging experiments were performed for four independent colonies per yeast strain. More than 85 patches were analyzed per strain, which corresponds to a statistical power of 98% to detect effects in patch lifespan of 5 seconds. For other similar studies see Kaksonen et al., Cell (2005): https://doi.org/10.1016/j.cell.2005.09.024.

Data exclusions

During the cryo-EM image processing, particles that represented false picks or particles that did not contribute high-resolution information to the reconstructions were discarded through 2D and 3D classification procedures. This process, which is required to obtain high-resolution reconstructions, is a standard procedure in cryo-EM image processing.

In TIRF microscopy experiments determining actin filament depolymerization velocities, filaments that stick to the surface or exhibited prolonged pauses during depolymerization were excluded from the analysis. The latter is a well-known artefact resulting from photo-induced oligomerization, see for example Niedermeyer et al. PNAS (2012): https://doi.org/10.1073/pnas.1121381109.

For the live cell imaging experiments only patches where the full lifespan was captured within the movies were considered, and patches with a lifespan shorter than 4 seconds were not taken into account to avoid cable detection artifacts, see for example Planade, Belbahri et al., PLoS Biol., (2019): https://doi.org/10.1371/journal.pbio.3000500.

Replication

All cryo-EM datasets were collected in one session per structure and were not repeated. It is unattainable from a time and cost perspective to repeat cryo-EM data collection and processing on the exact same sample.

The in vitro bulk phosphate release assays were performed in triplicate. They were performed as independent experiments, with new protein aliquots from the same purification batch. All attempts at replication were successful.

The single filament microfluidic experiments were performed in replicates of 6 for wild-type actin and 12 for the N111S mutant. They were performed as independent experiments, with new protein aliquots from the same purification batch. All attempts at replication were successful.

The in vivo experiments were performed in duplicate for four independent colonies per strain. They were performed as independent experiments, growing new cultures of cells at different days. All attempts at replication were successful.

Randomization	For the 3D refinement of cryo-EM structures, particles were randomly split into two half sets. For all other experiments, randomization was not required because there were no confounding factors in our experiments that could have led to biased results. Covariates were not controlled.
Blinding	This study does not involve any experiments where blinding would be applicable, because there were no confounding factors in our experiments that could have led to biased results.

Behavioural & social sciences study design

All studies must disclose on these points even when the disclosure is negative.

Study description	*Briefly describe the study type including whether data are quantitative, qualitative, or mixed-methods (e.g. qualitative cross-sectional, quantitative experimental, mixed-methods case study).*
Research sample	*State the research sample (e.g. Harvard university undergraduates, villagers in rural India) and provide relevant demographic information (e.g. age, sex) and indicate whether the sample is representative. Provide a rationale for the study sample chosen. For studies involving existing datasets, please describe the dataset and source.*
Sampling strategy	*Describe the sampling procedure (e.g. random, snowball, stratified, convenience). Describe the statistical methods that were used to predetermine sample size OR if no sample-size calculation was performed, describe how sample sizes were chosen and provide a rationale for why these sample sizes are sufficient. For qualitative data, please indicate whether data saturation was considered, and what criteria were used to decide that no further sampling was needed.*
Data collection	*Provide details about the data collection procedure, including the instruments or devices used to record the data (e.g. pen and paper, computer, eye tracker, video or audio equipment) whether anyone was present besides the participant(s) and the researcher, and whether the researcher was blind to experimental condition and/or the study hypothesis during data collection.*
Timing	*Indicate the start and stop dates of data collection. If there is a gap between collection periods, state the dates for each sample cohort.*
Data exclusions	*If no data were excluded from the analyses, state so OR if data were excluded, provide the exact number of exclusions and the rationale behind them, indicating whether exclusion criteria were pre-established.*
Non-participation	*State how many participants dropped out/declined participation and the reason(s) given OR provide response rate OR state that no participants dropped out/declined participation.*
Randomization	*If participants were not allocated into experimental groups, state so OR describe how participants were allocated to groups, and if allocation was not random, describe how covariates were controlled.*

Ecological, evolutionary & environmental sciences study design

All studies must disclose on these points even when the disclosure is negative.

Study description	*Briefly describe the study. For quantitative data include treatment factors and interactions, design structure (e.g. factorial, nested, hierarchical), nature and number of experimental units and replicates.*
Research sample	*Describe the research sample (e.g. a group of tagged Passer domesticus, all Stenocereus thurberi within Organ Pipe Cactus National Monument), and provide a rationale for the sample choice. When relevant, describe the organism taxa, source, sex, age range and any manipulations. State what population the sample is meant to represent when applicable. For studies involving existing datasets, describe the data and its source.*
Sampling strategy	*Note the sampling procedure. Describe the statistical methods that were used to predetermine sample size OR if no sample-size calculation was performed, describe how sample sizes were chosen and provide a rationale for why these sample sizes are sufficient.*
Data collection	*Describe the data collection procedure, including who recorded the data and how.*
Timing and spatial scale	*Indicate the start and stop dates of data collection, noting the frequency and periodicity of sampling and providing a rationale for these choices. If there is a gap between collection periods, state the dates for each sample cohort. Specify the spatial scale from which the data are taken*

Data exclusions	*If no data were excluded from the analyses, state so OR if data were excluded, describe the exclusions and the rationale behind them, indicating whether exclusion criteria were pre-established.*
Reproducibility	*Describe the measures taken to verify the reproducibility of experimental findings. For each experiment, note whether any attempts to repeat the experiment failed OR state that all attempts to repeat the experiment were successful.*
Randomization	*Describe how samples/organisms/participants were allocated into groups. If allocation was not random, describe how covariates were controlled. If this is not relevant to your study, explain why.*
Blinding	*Describe the extent of blinding used during data acquisition and analysis. If blinding was not possible, describe why OR explain why blinding was not relevant to your study.*

Did the study involve field work? ☐ Yes ☒ No

Reporting for specific materials, systems and methods

We require information from authors about some types of materials, experimental systems and methods used in many studies. Here, indicate whether each material, system or method listed is relevant to your study. If you are not sure if a list item applies to your research, read the appropriate section before selecting a response.

Materials & experimental systems

n/a	Involved in the study
☒	☐ Antibodies
☐	☒ Eukaryotic cell lines
☒	☐ Palaeontology and archaeology
☒	☐ Animals and other organisms
☒	☐ Human research participants
☒	☐ Clinical data
☒	☐ Dual use research of concern

Methods

n/a	Involved in the study
☒	☐ ChIP-seq
☒	☐ Flow cytometry
☒	☐ MRI-based neuroimaging

Eukaryotic cell lines

Policy information about cell lines

Cell line source(s)	BTI-Tnao38, species of origin - Trichoplusia ni. The cells were provided by S. Wohlgemuth and A. Musacchio (MPI Dortmund, Germany). The Musacchio lab obtained the cell line from G. Blissard (Cornell University, NY, USA) in 2011 - see Hashimoto et al. BMC Biotechnol. (2012) - doi: 10.1186/1472-6750-12-12. The BTI-Tnao38 cell line was used in our study for the production of recombinant β-actin variants.
Authentication	The BTI-Tnao38 cell line was not authenticated
Mycoplasma contamination	The BTI-Tnao38 cell line was not tested for mycoplasma contamination.
Commonly misidentified lines (See ICLAC register)	Research Resource Identifier: CVCL_Z252.
